# First‐Principles Investigation of Adsorption of Ethene on a Twice Oxidized NiF_2_ (001) Surface: A Model for the Simons Process

**DOI:** 10.1002/cphc.202500117

**Published:** 2025-12-16

**Authors:** Tilen Lindič, Jasmin Ahmad, Beate Paulus

**Affiliations:** ^1^ Institut für Chemie und Biochemie Freie Universität Berlin Arnimallee 22 14195 Berlin Germany

**Keywords:** adsorption study, electrochemical fluorination, fluorination of ethene, periodic density, functional, theory, Simons process

## Abstract

Electrochemical fluorination on a nickel anode (Simons process) is an important process for producing fluorinated compounds. Despite its success, the mechanism is still under debate. Here a first‐principles study is presented of fluorination of ethene on a model fluorinated (001) NiF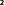 surface, which is chosen because it is stabilized under the external potential close to that at which the Simons cell operates and because it has a readily available ${\left[F_{2}\right]}^{-}$ unit providing fluorine source to aid fluorination reactions. The adsorption of the simplest double bond containing hydrocarbon on this surface is investigated. It is placed on the surface in different orientation, leading to six distinct structural outcomes upon relaxation. These include formation of 1,2‐difluoroethane, fluoroethene, and 1,2‐difluoroethene, alongside other fluorinated products as well as monocarbon fragments. This is one of the first computational studies of the catalytic Simons‐type fluorination and can, despite its simplicity, offers some insight into reaction pathways and surface interactions.

## Graphical abstract

A density functional theory study of ethene adsorption on a twice oxidized ${\text{NiF}}_{2}$ (001) surface reveals multiple fluorination pathways, providing insight into the catalytic role of the higher valent nickel surface in the Simons process (electrochemical fluorination on a nickel anode).

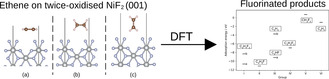

## 1 Introduction

Fluorination of different chemical compounds is an important area of research, because fluorinated compounds are ubiquitous in modern society, whether it is pharmaceuticals,^[^ ^1^, ^2^, ^3^ ^]^ agrochemicals,^[^ ^4^, ^5^ ^]^ fire retardants,^[^ ^6^ ^]^ or any other use. The need for efficient synthesis of these compounds on both a laboratory and industry scale is therefore needed. Electrochemical synthesis offers not only an inexpensive but also a sustainable synthetic path.^[^ ^7^ ^]^ One of the electrochemical fluorination techniques is fluorination on a nickel anode, also known as the Simons process.

It was first reported by Simons in 1949 in a series of articles.^[^ ^8^, ^9^, ^10^, ^11^, ^12^ ^]^ A year later, it was patented^[^ ^13^ ^]^ and has been used on an industrial scale ever since. The setup of the Simons process consists of a nickel anode immersed in anhydrous hydrogen fluoride. After the external potential is applied, the desired substrate to be fluorinated is added and the fluorination proceeds readily. Despite the general success and the age of the process, its mechanism is not completely understood and many different suggestions have been made over the years. One of the main proposed mechanisms is the radical cation mechanism (also referred to as the EC_b_EC_*N*_ mechanism, where each letter describes a stage in the mechanism).^[^ ^14^, ^15^, ^16^ ^]^ Despite some success, this mechanism does not explain why the fluorination occurs even in the case when the electrochemical cell is pre‐electrolyzed and the substrate is added after the discontinuation of the external potential.^[^ ^17^, ^18^ ^]^ This can, however, be described by the second proposed mechanism, which is believed to involve higher valent nickel fluorides, which are catalytically active.^[^ ^19^ ^]^ In this mechanism, higher valent ${\text{Ni}}_{x}F_{y}$ species are believed to be formed on the anode under the application of external potential. The fluorine bound to these nickel centers acts as a fluorinating agent when the substrate is added to the electrochemical cell. Over the years, ample experimental studies indirectly backed up this mechanism.^[^ ^20^, ^21^, ^22^, ^23^, ^24^ ^]^ Nevertheless, the exact nature of the formed ${\text{Ni}}_{x}F_{y}$ species is not known. It has been speculated that it could be ${\text{NiF}}_{3}$ doped with ${\text{NiF}}_{4}$ based on the high fluorination ability reported for these compounds.^[^ ^25^, ^26^ ^]^ A recent in situ X‐ray adsorption near edge structure study of the anode provided very concrete evidence that higher valent nickel centers are indeed present on the anode at applied potentials.^[^ ^27^ ^]^

To model a Simons‐type fluorination, we have identified a suitable surface cut with high‐valent nickel centers. It is a twice oxidized (001) surface derived from ${\text{NiF}}_{2}$. Previously, we have already published adsorption studies of ${\text{CH}}_{4}$ ^[^ ^28^ ^]^ and CO on this surface. In the present article, we report a first‐principles study of the adsorption of ethene.

With ethene, there are many possibilities of how it can be fluorinated; four hydrogen atoms can be substituted and there is also the possibility of reducing the double bond to a single C—C bond. Simons‐type fluorination of ethene has been reported, to the best of our knowledge, only once in the literature.^[^ ^29^ ^]^ In the experimental study, the authors observed the formation of multiple different products, predominantly perfluorinated, and partially fluorinated ethane. In addition, tetrafluoromethane, ethane and an unidentified compound were also observed. The ratio of these products depended on the experimental conditions. In the present study, our aim is to elucidate the individual steps in this catalytic process. As evidenced by experiments showing that the fluorination step of the Simons process still takes place even if the external potential is discontinued after the pre‐electrolysis of the cell (vide supra), we have deliberately not simulated external potential in our study. By doing so, we were able to study only the catalytic role of the nickel fluoride, which is the active fluorination species.^[^ ^27^ ^]^

## 2 Computational Details

All the results presented in this article were obtained using periodic spin‐unrestricted density functional theory (DFT) as implemented in the Vienna Ab initio Simulation Package (VASP), version 5.4.4.^[^ ^30^, ^31^, ^32^ ^]^ The Perdew–Burke–Ernzerhof (PBE) exchange correlation functional^[^ ^33^ ^]^ was used, as it was previously validated to adequately describe similar systems. Long‐range interactions were accounted for by adding Grimme's D3 dispersion correction^[^ ^34^ ^]^ with Becke‐Johnson damping^[^ ^35^ ^]^ on top of the PBE functional. Additionally, a Hubbard correction following the Dudarev approach,^[^ ^36^ ^]^ with an effective *U* value of 5.3 eV was employed. For all surface calculations, the first Brillouin zone was sampled using a Γ centered Monkhorst–Pack K‐point grid with the 8 × 8 × 1 mesh. Plane waves with projector‐augmented wave (PAW) potentials^[^ ^37^, ^38^ ^]^ were used as a basis set. Nickel 3d and 4s orbitals, as well as 2s and 2p orbitals of the other atoms, were treated explicitly, with all the other orbitals implicitly included in the core. All plane waves with kinetic energy up to 700 eV were included. Gaussian smearing with 0.10 eV as smearing width was used in all calculations.

The electronic convergence criterion was set to 10^−6^ eV for structural relaxation and single‐point energy calculations, and to 10^−8^ eV for the calculation of vibrational frequencies. The conjugate gradient algorithm was used for ionic relaxation with the convergence of the forces set to 0.01 eV Å^−1^. The shape and volume of the cells were kept fixed during relaxation, with only the ions allowed to move. The finite difference approach with the step width of 0.02 Å was used for the calculation of the vibrational frequencies. The free molecules were simulated in a cubic box of 15 Å. To locate possible transition states, the climbing image nudged elastic band (CI‐NEB) method was employed.^[^ ^39^, ^40^ ^]^ Bader charge analysis was performed with an external code of the Henkelman group.^[^ ^41^ ^]^ All the structures were visualized with VESTA.^[^ ^42^ ^]^

### 2.1 Structural Model

A twice oxidized (001) ${\text{NiF}}_{2}$ surface was chosen as a suitable model for modeling Simons‐type fluorination. As this surface was described in detail in our previous study,^[^ ^28^ ^]^ only a brief description will be given here. It is a nonstoichiometric cut of the (001) ${\text{NiF}}_{2}$ surface, which possesses a $F_{2}$ unit (hence it can be termed twice oxidized and we label it as ${\text{NiF}}_{2}{\text{(F}}_{2})$ surface), which is readily available to aid the fluorination reaction. In a previous study by our group, we have shown that in general the stoichiometric ${\text{NiF}}_{2}$ surfaces are more stable. However, fluorine rich surfaces were calculated to be stabilized under external potential.^[^ ^43^ ^]^ Particularly the twice oxidized ${\text{NiF}}_{2}{\text{(F}}_{2})$ surface was shown to be stable at the operating potentials of the Simons process.^[^ ^28^ ^]^ The formal oxidation state of the nickel centers of the stoichiometric surface would be +2, but because two fluorine atoms are attached, the formal oxidation state of nickel would be +4. However, the calculated magnetic moments of the surface nickel atoms (2.218 *μ*_B_) point to a nickel in the oxidation state of +3. The calculated magnetic moments on each of the surface fluorine atoms are 0.595 *μ*_B_, corresponding to about one negative charge on the adsorbed $F_{2}$. Therefore, surface fluorine atoms can be thought of as an ${{\text{[F}}_{2}\text{]}}^{-}$ unit. This is also reflected in the Ni—F bond lengths, which are significantly shorter on the surface (1.881 Å) compared to the bulk region (2.014 Å). Three main characteristics making these surfaces suitable as a model for Simons‐type fluorination are: 1) higher valent nickel on the surface; 2) ${{\text{[F}}_{2}\text{]}}^{-}$ unit as a fluorine source; and 3) enhanced stability at the operational potentials of the Simons process.

**Figure 1:**
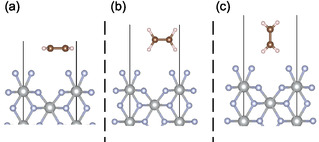
Different possible starting orientations of ethene along its three C_2_ axes on ${\text{NiF}}_{2}{\text{(F}}_{2})$ (001) surface. a) flat on the surface, b) perpendicular to the surface along the longer edge, and c) perpendicular to the surface along the shorter edge. Ni atoms are shown in gray, *F* in blue, *C* in brown, and *H* in white.

Six different adsorption sites of higher symmetry were identified on the surface (see Figure S1, Supporting information). Ethene molecule was placed on each site (in such a way that its center of mass was aligned with the adsorption site) in three different orientations (see **Figure** **1**; note that for clarity only the upper part of the slab model is shown in this and other figures in the manuscript.) along its three C_2_ rotational axes; lying flat on the surface and perpendicular to the surface plane facing it with either the longer or the shorter edge, respectively. Because various problems were encountered with the starting configurations where ethene was place perpendicular to the surface, facing it with the shorter edge, the ethene molecule was tilted by 5° and 10°, respectively. Three distances from the surface were investigated for each of the starting configurations, 1.7, 2.2, and 3.0 Å. These correspond to the distance between the topmost part of the surface (i.e., the plane of the ${{\text{[F}}_{2}\text{]}}^{-}$ unit) and the closest atom of the ethene molecule. In total 96 different starting configurations were created (for a more detailed explanation, see Supporting Information).

Adsorption energies were calculated by taking the free surface and adsorbate as reference points in the following way.

(1) $$E_{\text{ads}}=E_{\text{surf}+\text{ads}}-(E_{\text{surf}}+2E_{\text{ethene}})$$

*E*_ads_ is the adsorption energy, *E*_surf+ads_ is the energy of the combined surface and adsorbate system (after structural relaxation), *E*_surf_ is the energy of a clean ${\text{NiF}}_{2}{\text{(F}}_{2})$ surface, and *E*_ethene_ is the energy of a free ethene molecule (note that coefficient 2 is there because the ethene was adsorbed symmetrically on both sides of the slab to generate a symmetric slab model).

Charge transfer is defined via Bader charges by

(2) $$\Delta q_{i}=q_{i\text{,free}}-q_{i\text{,surface}+\text{ethene}}$$

Δ*q*_*i*_ is the charge transfer of atom *i*, *q*_*i*,free_ is the Bader charge of atom *i* in the free system (i.e., a clean surface or a free molecule) and *q*_*i*,surface+ads_ is the Bader charge of the *i*‐th atom calculated after the adsorption of ethene on the surface. Defining the charge transfer in this manner, the positive values correspond to the gain of electron density and the negative values correspond to its loss.

## 3 Results and Discussion

**Figure 2:**
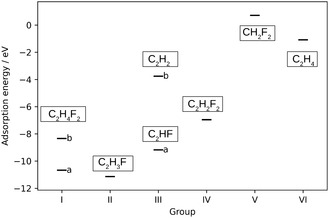
The lowest adsorption energies and the main outcomes of the adsorption of ethene on the (001) ${\text{NiF}}_{2}{\text{(F}}_{2})$ surface grouped into six different groups (and their subgroups, where applicable).

The ethene molecule was placed on the surface at the six positions of higher symmetry along its three C_2_ axes at three different distances, 1.7, 2.2, and 3.0 Å away from the surface. In total, 96 different starting configurations were created in such a way. Upon structural relaxation, the results were grouped into six different groups depending on the resulting outcome on the surface (see **Figure** **2**). We have based this classification on the chemical changes that the ethene molecule undergoes after structural relaxation. This allows us to systematically discuss the various pathways occurring on the surface.

In the first group, the ethene molecule was fluorinated, resulting in the formation of 1,2‐difluoroethane. In the second group, one hydrogen atom of ethene was substituted by fluorine, forming a fluoroethene. In the third group, the double bond was reduced into a triple bond, forming either ethyne or fluoroethyne. Group IV consists of outcomes where two of the hydrogen atoms of ethene were substituted by fluorine and 1,2‐difluoroethene was formed on the surface. Group V is somewhat special because there the C—C bond was broken and only species consisting of one carbon atom were formed. In the last, sixth group, the ethene molecule remained weakly physisorbed on the surface. All the numerical data are collected in the Supporting information, and here only the most stable structures of each group, together with any apparent emerging trends, will be discussed.

### 3.1 Group I

**Figure 3:**
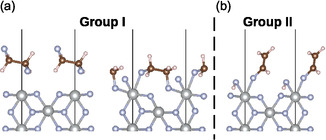
Structures of the most stable configurations of groups I (a), I (b) and II after structural relaxation on the (001) ${\text{NiF}}_{2}{\text{(F}}_{2})$ surface. The group number is labeled above the structure and (where applicable) the subgroup below each respective structure. Ni atoms are represented in gray, fluorine atoms in blue, carbon atoms in brown and hydrogen atoms in white.

In group I, there are 35 structures, where the ethene molecule was doubly fluorinated and 1,2‐difluoroethane was formed on the surface. This group can be further divided into two subgroups (energetically the most stable structures of both subgroups are shown in **Figure** **3**); (a) where the two fluorine atoms come from the surface ${{\text{[F}}_{2}\text{]}}^{-}$ unit and (b) where one of the fluorine atoms comes from the surface ${{\text{[F}}_{2}\text{]}}^{-}$ unit and the other one from one of the fluorine atoms which is in the plane of the surface nickel. The adsorption energies of subgroup (a) lie in the range from −10.67 to −9.77 eV and comprise 32 structures. Adsorption energies of the three structures of subgroup (b) are almost the same for all three of them at −8.34 eV.

The most stable configuration in subgroup I (a) is the gauche 1,2‐difluoroethane. Its starting configuration was that of the ethene molecule placed on the surface perpendicularly with the longer edge facing the surface. Some selected structural parameters for this structure are collected in **Table** **1**. All the structural parameters are very close to those calculated for the free gauche 1,2‐difluoroethane (C—C bond length of 1.503 Å, C—F of 1.408 Å, and two C—H bond distances of 1.100 and 1.102 Å, respectively). The slight deviation observed (for the most stable configuration, i.e., slight shortening of the C—C bond and distortion for other bonds that are not equal on both the carbons) can be contributed to the interaction with the surface. No apparent trend about the conformation or the starting configuration can be elucidated from the formed structures on the surface.

**Table 1:** Adsorption energies (*E*_ads_ in eV) and selected structural parameters (*d* in Å) for group I and II structures.

Group	*E*_ads_	d(CC)	d(CF)	d(CF)	d(CH)	d(CH)	d(CH)	d(CH)	
I (a)	−10.67	1.487	1.454	1.420	1.095	1.098	1.097	1.102	–
I (b)	−8.34	1.461	1.485	1.432	1.085	1.092	1.092	1.101	–
Group	*E*_ads_	d(CC)	d(CF)	d(CH)	d(CH)	d(CH)	d(NiF)	d(NiF)	d(HF)
II	−11.14	1.332	1.388	1.087	1.088	1.088	2.057	2.190	1.047

**Table** **2** presents the values for the magnetic moment as well as charge transfer on the surface nickel and charge transfer on the ${{\text{[F}}_{2}\text{]}}^{-}$ unit and on the atoms originating from ethene for the most stable structure in subgroup I a). Similar trends for these values are observed for other structures in the group. The magnitude of the magnetic moment on the surface Ni atom (around 1.8 *μ*_B_ ) indicates nickel in the oxidation state +2. This is also in agreement with the positive value of charge transfer (around +0.3 e), indicating a gain of electron density and transition of the formerly Ni(III/IV) in the clean slab to Ni(II). This is a consequence of loss of ${{\text{[F}}_{2}\text{]}}^{-}$ unit (the resulting surface without the adsorbate can be thought of as the stoichiometric (001) ${\text{NiF}}_{2}$ surface). The charge transfer on the surface fluorine atoms is positive (around +0.3 to +0.4 e), indicating the gain of electron density, which can be explained by structural changes during the structural relaxation. Both of these two fluorine atoms are connected to carbon, from which they are able to withdraw electron density. This is further corroborated with the highly negative charge transfer values on both carbon atoms (around −0.5 e). The charge transfer values on the hydrogen atoms are slightly negative, which is a consequence of the highly electronegative fluorine atoms now being bound to the same carbon atoms. Thus, they withdraw some of the electron density from the hydrogen atoms.

**Table 2:** Magnetic moment on the surface nickel (*μ* in $\mu_{B}$) and charge transfer (Δ*q* in e) on surface Ni and F atoms as well as C and H atoms of adsorbed ethene for the most stable structures of groups I and II.

Group	*μ*(Ni)	Δ*q*(Ni)	Δ*q*(F)	Δ*q*(F)	Δ*q*(C)	Δ*q*(C)	Δ*q*(H)	Δ*q*(H)	Δ*q*(H)	Δ*q*(H)
I (a)	−1.818	0.295	0.349	0.374	−0.455	−0.528	−0.072	−0.096	−0.034	−0.075
I (b)	−1.812	0.309	0.353	0.471	−0.410	−0.488	−0.114	−0.133	−0.000	−0.129
II	−1.828	0.283	0.359	0.490	−0.461	0.006	−0.143	−0.699	−0.037	−0.045

In the subgroup (b) of group I, there are three structures. The main difference compared to the subgroup (a) is the origin of the fluorine atoms. These structures could best be described as a chemisorbed ethene molecule through one of the surface fluorine atoms and one of the fluorine atoms in the plane of the surface nickel (see Figure 3), effectively still forming 1,2‐difluoroethane. Compared to the free 1,2‐difluoroethane molecule, these three structures are slightly more distorted than those in subgroup (a). However, despite this distortion, the structural parameters are still very close to those of free 1,2‐difluoroethane. The same as for group I (a), the surface nickel atoms are in the oxidation state +2, and the charge pattern is very similar as discussed above.

### 3.2 Group II

The most stable adsorption among all groups is found in group II, the corresponding structure is shown in Figure 3. Structures in this group correspond to the formation of fluoroethene, where one of the hydrogen atoms was substituted with fluorine. The remaining hydrogen then migrated to the remaining surface fluorine atom, forming HF, which in almost all cases stayed adsorbed to the surface nickel atom. In total, there are 37 different structures in this group, with the adsorption energies in the range from −11.14 to −9.16 eV. Some selected structural parameters of the most stable configuration are shown in Table 1 (all other structures have similar values and are collected in the Supporting information). The C—C bond length at 1.322 Å is very close to the free fluoroethene molecule (1.326 Å). The three C—H bond distances also do not deviate from the free molecule (1.090, 1.087 and 1.088 Å). The largest deviation compared to the free molecule can be seen for the C—F bond distance (1.363 Å in free molecule). The reason for this longer C—F bond is the proximity of fluorine to Ni with its attractive interaction. The HF bond distance is elongated compared to the free molecule (0.938 Å), which is also a consequence of the interaction with the surface nickel atom.

The magnitude of the magnetic moment on the surface of nickel (see Table 2 for the most stable structure) points to nickel in oxidation state +2. This is also supported by a positive charge transfer value of around +0.3 e, which indicates a gain of electron density. Magnetic moments on the surface fluorine atoms and those atoms originating from the ethene molecule are 0 *μ*_B_. Same trend is observed for all other structures in the group. Charge transfer on the fluorine atoms originating from the surface ${{\text{[F}}_{2}\text{]}}^{-}$ unit takes two distinct values, one around +0.4 e and another around +0.5 e. This can be explained by looking at the final structure. The larger value corresponds to the fluorine atom that accepts the hydrogen atom, and the lower value corresponds to the fluorine which connects to the carbon. Likewise, two distinct values can be seen for the two carbon atoms. One is around −0.5 e and corresponds to the carbon to which the fluorine atom attaches. Because of the way we define charge transfer, this can be explained by the electronegativity of the fluorine atom, which abstracts much more electron density from carbon than hydrogen. The other carbon, which is still in roughly the same chemical environment (i.e., connected to two hydrogen atoms) as in the free molecule, has a negligible charge transfer. A similar trend for the charge transfer can be elucidated for the hydrogen atoms originating from the adsorbed ethene molecule. The one hydrogen atom that detaches from carbon and forms an HF molecule with one of the surface fluorine atoms has a highly negative charge transfer (around −0.7 e) because the fluorine atom abstracts electron density from it. Two of the hydrogen atoms bound to the same carbon have negligible values of charge transfer, whereas the remaining hydrogen atom bound to the carbon, to which also fluorine is bound, has a charge transfer in the range from −0.1 to −0.2 e.

### 3.3 Group III

In group III, there are only two structures and both correspond to the reduction of the ethene double bond to a triple bond. Despite the fact that these products are not observed in the experiment, we believe that at the level of our model, they might still be important. As the Simons process occurs in anhydrous HF and under electrochemical conditions, it can well be possible that these species would react further, giving products observed in the experiment.

**Figure 4:**
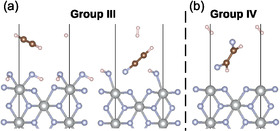
Structures of the most stable configurations of groups III (a), III (b) and IV after structural relaxation on the (001) ${\text{NiF}}_{2}{\text{(F}}_{2})$ surface. The group number is labeled above the structure and (where applicable) the subgroup below each respective structure. Ni atoms are represented in gray, fluorine atoms in blue, carbon atoms in brown and hydrogen atoms in white.

Both structures of group III are shown in **Figure** **4**. Ethene placed on the surface perpendicularly along the longer edge was the starting configuration for both structures. The structure labeled a) corresponds to the formation of ethyne. Its adsorption energy is −9.181 eV. Some of its selected structural parameters are collected in **Table** **3**. Both the C—C bond length and the two C—H bond lengths are very close to the free molecule (1.206 and 1.070 Å, respectively), which can be expected since the ethyne molecule is desorbed from the surface. Two hydrogen atoms from adsorbed ethene migrated to the two surface fluorine atoms, effectively forming two adsorbed HF molecules. The two Ni—F bond lengths are significantly elongated compared to the clean surface (1.881 Å) and are much closer to the Ni—F bond distances characteristic of the Ni(II) centers found in the bulk region (around 2 Å). This is also reflected in the magnitude of the magnetic moment of the surface nickel atom (1.8 *μ*_B_; see **Table** **4**) and the positive charge transfer which both indicate a nickel atom in the oxidation state +2. Charge transfer on both the surface fluorine atoms is highly positive (around +0.5 e) because they gain electron density from the hydrogen atoms that attach to them. In turn, those have a highly negative charge transfer (around −0.7 e). The second outcome in this group (denoted as b) in Figure 4) is the formation of fluoroethyne and a hydrogen molecule. The remaining hydrogen from ethene is adsorbed through one of the surface fluorine atoms, similarly to that in subgroup (a), forming an HF molecule. The adsorption energy of this structure is much higher, compared to the subgroup (a), at −3.76 eV, due to the artificial formation of $H_{2}$ within our model. All structural parameters of fluoroethyne (see Table 3) are comparable to those of the free molecule (C—C bond length at 1.202 Å, C—H at 1.068 Å, and C—F at 1.290 Å). Magnetic moment and charge transfer on the surface of nickel (see Table 4) point to the nickel in the oxidation state +2. Charge transfer on other atoms follows a trend similar to that described for the structure in subgroup (a), with the notable exception of the carbon atoms, which in this case show negative charge transfer. This can be contributed to the fact that now one of the hydrogen atoms has been exchanged by fluorine, which draws out the electron density from carbon.

**Table 3:** Adsorption energies (*E*_ads_ in eV) and selected structural parameters (*d* in Å) for group III and IV structures.

Group	*E*_ads_	d(CC)	d(CH)	d(CH/F)	d(NiF)	d(NiF)	d(HF)	d(HF/H)
III (a)	−9.18	1.208	1.071	1.071	2.038	2.037	0.992	0.991
III (b)	−3.76	1.208	1.068	1.275	2.149	–	0.968	0.751
Group	*E*_ads_	d(CC)	d(CF)	d(CF)	d(CH)	d(CH)	d(HH)	–
IV	−6.96	1.327	1.385	1.358	1.089	1.088	0.751	–

**Table 4:** Magnetic moment on the surface nickel (*μ* in $\mu_{B}$) and charge transfer (Δ*q* in e) on surface Ni and F atoms as well as C and H atoms of adsorbed ethene for the most stable structures of groups III and IV.

Group	*μ*(Ni)	Δ*q*(Ni)	Δ*q*(F)	Δ*q*(F)	Δ*q*(C)	Δ*q*(C)	Δ*q*(H)	Δ*q*(H)	Δ*q*(H)	Δ*q*(H)
III (a)	_−_−1.831	0.281	0.464	0.482	−0.011	0.305	−0.177	−0.182	−0.718	−0.717
III (b)	−1.816	0.304	0.343	0.474	−0.198	−0.365	0.040	0.020	−0.182	−0.672
IV	−1.816	0.291	0.377	0.384	−0.556	−0.481	0.026	0.026	−0.187	−0.116

### 3.4 Group IV

In group IV, there are four structures (the most stable one is shown in Figure 4) that correspond to the formation of 1,2‐difluoroethene and a hydrogen molecule. Their adsorption energies range from −6.96 to −4.36 eV and are, compared to group III (b), where also $H_{2}$ is formed, more stable. In all of their starting configurations, the ethene molecule was oriented perpendicular to the surface along its longer edge. In the lowest and two of the highest energy configurations, the *Z* isomer was formed, whereas in the energetically second‐most stable structure, the *E* isomer formed. In the case of the two structures that were the highest in energy, one of the fluorine atoms came from the surface, and the other one from the fluorine in the plane of the surface nickel. Similarly as for group III, these structures are not observed in the experiment but could nonetheless be important, as with the addition of HF, the double bond could be further fluorinated, yielding the observed experimental products.

Some selected structural parameters are shown in Table 3 and they are all very close to the free molecule and not much distortion is observed because both the 1,2‐difluoroethene and the hydrogen molecule are almost desorbed from the surface and hence the interaction with the surface is not strong. Similarly, as discussed previously for other groups, the magnetic moment and charge transfer on the surface nickel (see Table 4 indicate the oxidation state +2.

### 3.5 Group V

**Figure 5:**
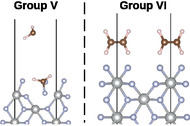
Structures of the most stable configurations of groups V and VI after structural relaxation on the (001) ${\text{NiF}}_{2}{\text{(F}}_{2})$ surface. The group number is labeled above the structure and (where applicable) the subgroup below each respective structure. Ni atoms are represented in gray, fluorine atoms in blue, carbon atoms in brown and hydrogen atoms in white.

Structures in group V are somewhat outliers compared to the other groups for two reasons. First, the C—C bond is broken and monocarbon species are formed, and second, all of their adsorption energies are positive. There are five structures in this group (the most stable one is shown in **Figure** **5**). In all structures, a difluoromethane and ${\text{CH}}_{2}$ carbene were formed. One of the fluorine atoms came from the surface, and the other one came from the fluorine in the plane of the surface nickel. These products were only formed on the surface when the starting orientation of the ethene was perpendicular to the surface along the shorter edge. Because the carbene fragment went quite high into the vacuum region, the vacuum size for these structures was increased in order to avoid any possible interactions between the top and the bottom layers of the surface (for a more detailed explanation, see Supporting information). The adsorption energies in this group are in the range from 0.72 to 2.03 eV. Positive values indicate that with respect to the clean surface and the free ethene, these outcomes are thermodynamically unfavorable. Despite this, the outcomes in this group are important because species containing only one carbon atom are observed in the experiment. Furthermore, in the actual Simons process, the ${\text{CH}}_{2}$ would quickly react with HF to form monofluorinated methane.

### 3.6 Group VI

In the last group, there are five structures where the ethene molecule only weakly interacts with the surface and stays structurally unchanged. Their adsorption energies are in the range from −1.08 to −0.18 eV. Structurally, no significant change can be observed for either the surface Ni—F bond lengths or the bond lengths of the adsorbed ethene molecule. The surface nickel atom remains in the same oxidation state as in the clean surface (see **Table** **5** for magnetic moment and charge transfer values). A slight positive value of charge transfer on the fluorine atoms and a slight negative value on the two hydrogen atoms closest to the surface indicate that there is, nevertheless, a very weak interaction between the adsorbed ethene and the surface.

**Table 5:** Magnetic moment on the surface nickel (*μ* in $\mu_{B}$) and charge transfer (Δ*q* in e) on surface Ni and F atoms as well as C and F atoms of adsorbed ethene for the most stable structures of groups V and VI.

Group	*μ*(Ni)	Δ*q*(Ni)	Δ*q*(F)	Δ*q*(F)	Δ*q*(C)	Δ*q*(C)	Δ*q*(H)	Δ*q*(H)	Δ*q*(H)	Δ*q*(H)
V	−1.819	0.304	0.447	0.380	0.006	−0.921	−0.195	−0.159	0.001	−0.007
VI	−2.229	0.006	0.205	0.224	−0.066	−0.032	0.012	−0.159	0.012	−0.159

### 3.7 Transition State Search

Within our model, the monofluorinated ethene is the most stable adsorption outcome on the surface. However, in the experiment, mainly perfluorinated ethane was observed. This discrepancy is a direct consequence of how our model is constructed, with only two fluorine atoms available (from the surface ${{\text{[F}}_{2}\text{]}}^{-}$ unit). In the experiment, where the Simons process takes place in anhydrous HF, there is a virtually unlimited fluorine source and further fluorination steps can occur, leading to the formation of perfluorinated products. To understand the possible mechanism leading to perfluorinated products, we focused on the transformation from fluoroethene to 1,2‐difluoroethane. This step is particularly interesting because, within our surface model, it involves the least fluorinated and the most fluorinated outcome on the surface. The initial and the final structure were chosen so that they were close, so that they had similar adsorption energies and were at similar positions on the surface.

**Figure 6:**
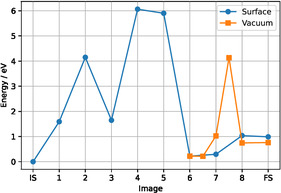
Energy profile of the NEB calculation for transformation from fluoroethene to 1,2‐difluoroethane with 8 images between the initial structure (IS) and the final structure (FS) shown in blue. In orange is shown the NEB calculation in vacuum for the transformation between the 1,1‐difluoroethane and 1,2‐difluoroethane in vacuum, with 4 images.

The energy profile of the NEB calculation is shown in **Figure** **6**. Between the initial state (IS) and the final state (FS), eight equidistant images were created. As can be seen from the energy profile, there are two peaks of high energy, at images 2 and 4. The activation barrier between the IS and image 2 is 4.15 eV. Calculation of vibrational frequencies for this image gave a single imaginary frequency at 348.15i cm^−1^. The second peak at image four is 6.06 eV higher in energy compared to the IS. It also has one imaginary frequency at 275.96i cm^−1^ (and a much smaller one at 26.89i cm^−1^), so it can also be described as a pure transition state from the viewpoint of theory.

**Figure 7:**
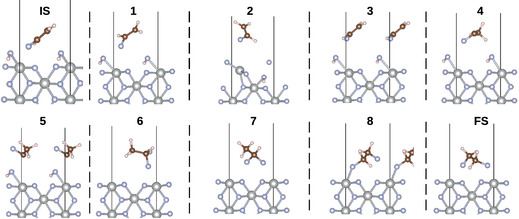
Structures of the initials state (IS), the final state (FS) and the eight calculated images in between (the number of each image is indicated above its structure). Ni atoms are represented in gray, fluorine atoms in blue, carbon atoms in brown and hydrogen atoms in white.

Structures of the initial and FS, as well as of the eight images in between, are shown in **Figure** **7**. The first three images represent mainly the rotation of fluoroethene. In the first image, the fluoroethene molecule is simply rotated and moved closer to the surface. Going to the second image, the hydrogen adsorbed through the surface fluorine atoms migrates to one of the fluorine atoms in the plane of the surface nickel. Then this hydrogen migrates back to the surface fluorine atom, together with the rotation of the fluoroethene molecule in the third image. The energy barrier for this step is quite high (cf. image 2 in Figure 6). One of the reasons for this could be the fact that this is modeled in a vacuum, but in a more realistic system, there would also be HF as a solvent, which would interact with the adsorbates.

In the fourth step, one of the hydrogen atoms of fluoroethene migrates to the other carbon. The resulting molecule approaches the surface with a C—C bond, which, compared to image 3 where it is 1.32 Å, is elongated to 1.46 Å. The angles between the C—C bond and the three hydrogen atoms are 110.5°, 102.8°, and 118.2°, respectively. This indicates that the previously *sp* 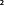 carbon atom is now closer to being *sp* 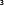 hybridized. The whole structure then rotates around the C—C axis in the fifth image to come closer to the adsorbed HF on the surface with the unsaturated carbon (i.e., the one with only one fluorine atom attached to it). In the sixth image, the HF molecule on the surface is split and added to the aforementioned carbon atom, forming 1,1‐difluoroethane. This is then further rotated in the seventh image. In the last image, the exchange between a fluorine and a hydrogen of different carbon atoms occurs, and then the structure is desorbed from the surface and rotated into the FS.

A second NEB calculation, shown in orange in Figure 6, is the transformation between 1,1‐difluoroethane and 1,2‐difluoroethane in vacuum, without surface. With this, we wanted to evaluate the surface effects on this path of the reaction. A quite high barrier in vacuum (almost 4 eV) indicates that the surface indeed lowers this barrier and aids the final isomerization.

The presence of two high energy points on this potential energy profile indicates a complex and multistep mechanism. It should be noted that the investigated reaction path does not represent the overall thermodynamic and kinetic progress of the Simons process. It can indicate that the paths toward different products that are observed in the Simons process might be independent of each other and do not undergo a stepwise fluorination pathway. Despite that, we were able to show how the double bond is reduced to a single bond and finally how the surface is involved in the final step of fluorination. In a more realistic process, probably the solvent (anhydrous HF) and the applied potential play a crucial role in lowering the reaction barrier.

## 4 Conclusions

The adsorption of ethene on a twice oxidized (001) ${\text{NiF}}_{2}\text{(F2)}$ surface, as a possible model for the Simons‐type fluorination, was investigated. After the ethene molecule was placed on the surface at different distances and in different orientations, the results of structural relaxation yielded a variety of different products. In most of the cases, 1,2‐difluoroethane was formed, which is also one of the products experimentally observed. In addition to breaking the double bond, fluorination of ethene can also occur, resulting in mono‐ and 1,2‐difluoroethene, depending on the starting configuration. In two cases, a double bond was reduced to a triple bond, with either ethyne or fluoroethyne as the outcome. In group IV, a double substitution of ethene was observed, resulting in the formation of 1,2‐difluoroethene. In cases where the C—C bond was broken, the formation of carbene was observed. This result is important because monocarbon species are observed in the experiment. Lastly, there are structures where there is only a weak interaction of ethene with the surface, which stays physisorbed. Magnetic moments and charge transfer were carefully analyzed to elucidate the change during the adsorption process. In all cases (except when the ethene molecule remained weakly physisorbed), the surface nickel atom was reduced from its initial oxidation state to the oxidation state +2.

A possible transition state search between fluoroethene and 1,2‐difluoroethane was investigated via CI‐NEB. Despite the high energy barrier, we were able to show how the adsorbate approaches the surface and how the surface fluorine atoms are involved in the catalytic process.

All in all, we have shown that even with such an elementary model as a surface of a binary nickel fluoride and the simplest double‐bond‐containing carbon‐based molecule, the outcomes on the surface are complex and nontrivial. To better describe the more realistic processes that take place during the Simons process, HF would have to be accounted for both as a solvent and as a fluorine source. Furthermore, the effects of external potential would also have to be taken into account.

## Conflict of Interest

The authors declare no conflict of interest.

## Supporting information

Supplementary Material

## Data Availability

The data that support the findings of this study are available in the supplementary material of this article.
