# Supplementary material for: First‐Principles Investigation of Adsorption of Ethene on a Twice Oxidized NiF2 (001) Surface: A Model for the Simons Process
Source: Chemphyschem. 2025 Dec 16;27(1):e202500117. doi: 10.1002/cphc.202500117 (PMC12810466; doi:10.1002/cphc.202500117)
Supplement: Supplementary file 1 — Supplementary Material [file CPHC-27-e202500117-s001.pdf]

Supporting Information

First-Principles Investigation of Ethene Adsorption on a Twice Oxidised NiF_2 (001) Surface: a Model for the Simons Process

T. Lindič, J. Ahmad and B. Paulus

List of Figures

Figure S1 Top view of the twice oxidised $NiF_2(F_2)$ (001) surface, with the adsorption sites labelled as circles in different colours and numbers in them. . . . 3
Figure S2 Structures after relaxation in group I. . . . 11
Figure S3 Structures after relaxation in group II. . . . 19
Figure S4 Structures after relaxation in group III. . . . 20
Figure S5 Structures after relaxation in group IV. . . . 21
Figure S6 Structures after relaxation in group V. . . . 24
Figure S7 Structures after relaxation in group VI. . . . 25

List of Tables

Table S1 Adsorption energies, and some selected structural parameters for the structures in group I. 4
Table S2 Magnetic moments on surface Ni, F and all atoms of ethene for group I. . . . 5
Table S3 Charge transfer on surface Ni, F and all atoms of ethene for group I. . . . 6
Table S4 Vibrational frequencies for the structures in group I. . . . 6
Table S5 Adsorption energies, and some selected structural parameters for the structures in group II. . . . 12
Table S6 Magnetic moments on surface Ni, F and all atoms of ethene for group II. . . . 12
Table S7 Charge transfer on surface Ni, F and all atoms of ethene for group II. . . . 13
Table S8 Vibrational frequencies for the structures in group I. . . . 14
Table S9 Adsorption energies, and some selected structural parameters for the structures in group III. . . . 20
Table S10 Magnetic moments on surface Ni, F and all atoms of ethene for group III. . . . 20
Table S11 Charge transfer on surface Ni, F and all atoms of ethene for group III. . . . 20
Table S12 Vibrational frequencies for the structures in group III. . . . 20
Table S13 Adsorption energies, and some selected structural parameters for the structures in group IV. . . . 21
Table S14 Magnetic moments on surface Ni, F and all atoms of ethene for group IV. . . . 21
Table S15 Charge transfer on surface Ni, F and all atoms of ethene for group IV. . . . 21
Table S16 Vibrational frequencies for the structures in group IV. . . . 22

Table S17 Adsorption energies, and some selected structural parameters for the structures in group V. 22
Table S18 Magnetic moments on surface Ni, F and all atoms of ethene for group V. 22
Table S19 Charge transfer on surface Ni, F and all atoms of ethene for group V. 22
Table S20 Vibrational frequencies for the structures in group V. 23
Table S21 Adsorption energies, and some selected structural parameters for the structures in group VI. 24
Table S22 Magnetic moments on surface Ni, F and all atoms of ethene for group VI. 25
Table S23 Charge transfer on surface Ni, F and all atoms of ethene for group VI. 25
Table S24 Vibrational frequencies for the structures in group VI. 25

1 General remarks

Numerical data and all the structures not directly presented in the main manuscript are collected in the following supporting information. To make the tables more readable the units are omitted and the following symbols are used:

- Orientation: initial orientation of the ethene molecule on the surface.
- Position: Number of the high symmetry position on the surface, on which the ethene molecule was placed on (see Figure S1).
- E_{ads}: adsorption energy in eV
- #: number of structure in each group (sorted by increasing adsorption energy)
- d(AB): bond distance between atom A and B, in Å.
- Magnetic moments are in the units of μ_B
- Charge transfer is in the units of e
- Vibrational frequencies in cm^{-1}, in case of imaginary frequency i is added after the number
- Nickel and fluorine atoms in the tables of magnetic moments and charge transfer correspond to the surface nickel atom and the fluorine atoms from the surface $[F_2]^-$ unit
- In figures of structures Ni atoms are shown in gray, F in blue, C in brown and H in white.

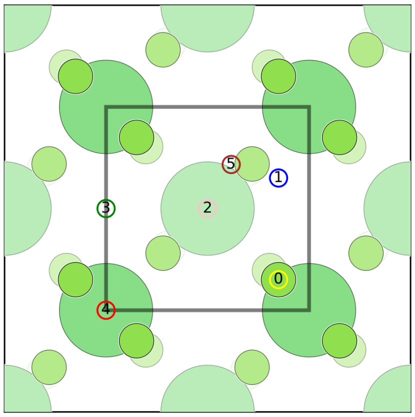

Figure S1: Top view of the twice oxidised $NiF_2(F_2)$ (001) surface, with the adsorption sites labelled as circles in different colours and numbers in them.

Main computational settings are already outlined in the main manuscript in the Computational part section. A few more practical points will be discussed here. The geometry relaxation was performed in three steps, In the first step only the ethene molecule was allowed to relax, in the second step the whole system was relaxed,

with convergence settings being set to 10^{-4} eV for electronic convergence and 0.02 eV/Å for the forces, finally the structures were relaxed with the parameters described in the main manuscript. This workflow was based on our previous experience which showed that if the structural relaxation is performed directly with the tightest convergence settings often times there is no convergence during the structural relaxation.

In our first attempted in this study we have set the parameter PREC in VASP to medium. This parameter sets the sizes of FFT grids and the accuracy of projectors in real space. Upon calculation vibrational frequencies we have observed some large or many small imaginary values. The reason for that was probably that the forces were not calculated accurately enough. We have then re-optimised those structures with the PREC parameter set to Accurate which led to better results (which are presented here). Furthermore, we have also repeated the whole workflow for those calculations.

For the orientation of ethene perpendicular to the surface with the shorter edge pointing to the surface we had to increase the vacuum size to 30 Å, because the molecule dissociated. With the usual vacuum size we used (20 Å) we encountered convergence problems, which were probably the consequence of the two fragments on the top and the bottom of the slab model to come too close together.

In the tables Orientation is marked in the following way: the first letter corresponds to the described orientations (f for flat on the surface, p for perpendicular with the longer edge facing the surface, t5 for tilted structures by 5°, t10 for tilted structures by 10°, f2 and p2 for the flat and perpendicular with the longer edge facing the surface structures which were re-optimised as described above, pv for the structures placed perpendicularly with shorter edge facing surface) the number after underscore indicates distance from the surface (1 corresponds to the ethene 1.7 Å away from the surface, 2 to 2.2 Å from the surface and 3 to ethene being 3.0 Å from the surface). Position indicated to which position on the surface ethene molecule was placed.

Overview of starting geometries

In total, 96 starting geometries were considered in the following way (for the labels see above):

3×f+3×p+2×t5+2×t10+3×f2+2×p2+pv = 16 configurations, which multiplied by 6 high-symmetry adsorption sites gives 96 starting structures.

2 Group I

Table S1: Adsorption energies, and some selected structural parameters for the structures in group I.

#	Orientation	Position	E_ads	d(CC)	d(CH)	d(CH)	d(CF)	d(CH)	d(CH)	d(CF)
1	p_3	0	-10.6719	1.487	1.095	1.098	1.454	1.097	1.102	1.420
2	p_1	1	-10.6659	1.487	1.097	1.102	1.420	1.095	1.098	1.454
3	p_3	4	-10.6400	1.486	1.096	1.102	1.421	1.095	1.098	1.460
4	p_1	5	-10.6285	1.490	1.096	1.096	1.450	1.098	1.099	1.418
5	t10_1	0	-10.5915	1.491	1.096	1.098	1.453	1.099	1.101	1.420
6	f2_2	5	-10.5824	1.492	1.100	1.102	1.420	1.097	1.098	1.449
7	f2_1	5	-10.5724	1.492	1.100	1.101	1.419	1.098	1.098	1.449
8	p_3	3	-10.3766	1.509	1.093	1.095	1.443	1.096	1.099	1.419
9	f_2	5	-10.1753	1.509	1.097	1.099	1.427	1.097	1.099	1.426

Continued on next page

Table S1: Table of Properties

#	Orientation	Position	E_{ads}	d(CC)	d(CH)	d(CH)	d(CF)	d(CH)	d(CH)	d(CF)
10	f_1	5	-10.1753	1.509	1.097	1.099	1.426	1.096	1.099	1.426
11	p_1	3	-10.1750	1.509	1.097	1.099	1.426	1.097	1.099	1.426
12	f2_3	2	-10.1459	1.510	1.097	1.099	1.426	1.097	1.099	1.426
13	f2_1	2	-10.1459	1.510	1.097	1.099	1.426	1.097	1.099	1.426
14	f2_2	2	-10.1456	1.510	1.097	1.099	1.426	1.097	1.099	1.426
15	f_3	3	-10.0576	1.496	1.098	1.102	1.418	1.098	1.102	1.418
16	f_1	3	-10.0283	1.494	1.098	1.101	1.422	1.098	1.101	1.422
17	f2_1	3	-10.0278	1.497	1.098	1.102	1.420	1.098	1.102	1.418
18	f_2	1	-10.0278	1.494	1.098	1.101	1.422	1.097	1.101	1.422
19	f2_2	3	-10.0241	1.497	1.098	1.102	1.420	1.098	1.102	1.418
20	f_2	2	-9.8577	1.513	1.093	1.099	1.425	1.093	1.099	1.425
21	f_1	2	-9.8576	1.513	1.092	1.099	1.425	1.092	1.099	1.425
22	f_3	2	-9.8576	1.513	1.093	1.099	1.425	1.093	1.099	1.425
23	p_1	2	-9.8575	1.513	1.092	1.099	1.426	1.092	1.099	1.426
24	p_2	2	-9.8573	1.513	1.093	1.099	1.426	1.093	1.099	1.426
25	f2_2	1	-9.8286	1.514	1.093	1.100	1.425	1.093	1.099	1.425
26	f2_1	1	-9.8285	1.514	1.093	1.099	1.425	1.093	1.099	1.425
27	f2_3	1	-9.8284	1.514	1.093	1.099	1.424	1.093	1.099	1.425
28	f_1	4	-9.7914	1.497	1.100	1.102	1.408	1.100	1.102	1.408
29	f_3	4	-9.7912	1.497	1.100	1.102	1.409	1.100	1.102	1.409
30	f_2	4	-9.7910	1.497	1.100	1.103	1.408	1.100	1.103	1.408
31	f2_1	4	-9.7658	1.498	1.101	1.103	1.409	1.101	1.103	1.409
32	f2_3	4	-9.7650	1.498	1.101	1.103	1.408	1.100	1.103	1.409
33	p_3	1	-8.3376	1.461	1.085	1.092	1.485	1.092	1.101	1.432
34	f_3	1	-8.3373	1.461	1.085	1.092	1.486	1.092	1.101	1.432
35	f_1	1	-8.3371	1.461	1.085	1.092	1.486	1.092	1.101	1.432

Table S2: Magnetic moments on surface Ni, F and all atoms of ethene for group I.

Ion/Position	Ion	Ion	Ni	F	F	C	C	H	H	H	H
1	p_3	0	-1.818	-0.010	-0.000	-0.000	0.000	-0.000	-0.000	0.000	0.000
2	p_1	1	-1.818	0.000	-0.009	0.000	-0.000	0.000	0.000	-0.000	-0.000
3	p_3	4	-1.816	-0.000	-0.012	0.000	-0.000	0.000	0.000	-0.000	-0.000
4	p_1	5	-1.818	0.000	-0.010	-0.000	-0.000	-0.000	-0.000	-0.000	-0.000
5	t10_1	0	-1.815	-0.013	0.000	0.000	-0.000	-0.000	-0.000	0.000	-0.000
6	f2_2	5	-1.817	0.000	-0.009	-0.000	-0.000	-0.000	-0.000	-0.000	0.000
7	f2_1	5	-1.817	0.000	-0.009	-0.000	-0.000	-0.000	-0.000	-0.000	0.000
8	p_3	3	-1.817	-0.009	-0.000	-0.000	-0.000	-0.000	-0.000	-0.000	0.000
9	f_2	5	-1.819	-0.006	-0.006	-0.000	-0.000	-0.000	0.000	0.000	-0.000
10	f_1	5	-1.819	-0.006	-0.006	-0.000	-0.000	-0.000	0.000	0.000	-0.000
11	p_1	3	-1.820	-0.006	-0.006	-0.000	-0.000	-0.000	0.000	-0.000	0.000
12	f2_3	2	-1.819	-0.006	-0.006	-0.000	-0.000	0.000	-0.000	0.000	-0.000
13	f2_1	2	-1.819	-0.006	-0.006	-0.000	-0.000	0.000	-0.000	0.000	-0.000
14	f2_2	2	-1.819	-0.006	-0.006	-0.000	-0.000	0.000	-0.000	0.000	-0.000
15	f_3	3	-1.818	-0.006	-0.006	-0.000	-0.000	-0.000	-0.000	-0.000	-0.000
16	f_1	3	-1.816	-0.009	-0.009	-0.000	-0.000	-0.000	-0.000	-0.000	-0.000
17	f2_1	3	-1.818	-0.007	-0.006	-0.000	-0.000	-0.000	-0.000	-0.000	-0.000
18	f_2	1	-1.816	-0.009	-0.009	-0.000	-0.000	-0.000	-0.000	-0.000	-0.000
19	f2_2	3	-1.818	-0.007	-0.006	-0.000	-0.000	-0.000	-0.000	-0.000	-0.000
20	f_2	2	-1.813	-0.003	-0.003	-0.000	-0.000	-0.000	-0.000	-0.000	-0.000
21	f_1	2	-1.813	-0.003	-0.003	-0.000	-0.000	-0.000	-0.000	-0.000	-0.000
22	f_3	2	-1.813	-0.003	-0.003	-0.000	-0.000	-0.000	-0.000	-0.000	-0.000
23	p_1	2	-1.813	-0.003	-0.003	-0.000	-0.000	-0.000	-0.000	-0.000	-0.000
24	p_2	2	-1.813	-0.003	-0.003	-0.000	-0.000	-0.000	-0.000	-0.000	-0.000
25	f2_2	1	-1.813	-0.003	-0.003	-0.000	-0.000	-0.000	-0.000	-0.000	-0.000
26	f2_1	1	-1.813	-0.003	-0.003	-0.000	-0.000	-0.000	-0.000	-0.000	-0.000
27	f2_3	1	-1.813	-0.003	-0.003	-0.000	-0.000	-0.000	-0.000	-0.000	-0.000
28	f_1	4	-1.814	-0.005	-0.005	-0.000	-0.000	0.000	-0.000	-0.000	0.000
29	f_3	4	-1.814	-0.005	-0.005	-0.000	-0.000	0.000	-0.000	-0.000	0.000

Continued on next page

Ion/Position	Ion	Ion	Ni	F	F	C	C	H	H	H	H
30	f_2	4	-1.814	-0.005	-0.005	-0.000	-0.000	0.000	-0.000	-0.000	0.000
31	f2_1	4	-1.814	-0.005	-0.005	-0.000	-0.000	-0.000	0.000	-0.000	0.000
32	f2_3	4	-1.814	-0.005	-0.006	-0.000	-0.000	-0.000	0.000	-0.000	0.000
33	p_3	1	-1.812	-0.013	-0.053	-0.001	-0.001	-0.000	-0.000	-0.001	0.000
34	f_3	1	-1.812	-0.013	-0.053	-0.001	-0.001	-0.000	-0.000	-0.001	0.000
35	f_1	1	-1.812	-0.013	-0.053	-0.001	-0.001	-0.000	-0.000	-0.001	0.000

Table S3: Charge transfer on surface Ni, F and all atoms of ethene for group I.

Ion/Position	Orientation	Position	Ni	F	F	C	C	H	H	H	H
1	p_3	0	0.295	0.349	0.374	-0.455	-0.528	-0.072	-0.096	-0.034	-0.075
2	p_1	1	0.292	0.357	0.369	-0.504	-0.458	-0.062	-0.092	-0.069	-0.079
3	p_3	4	0.299	0.351	0.363	-0.508	-0.444	-0.050	-0.088	-0.073	-0.094
4	p_1	5	0.297	0.354	0.372	-0.447	-0.528	-0.081	-0.077	-0.065	-0.066
5	t10_1	0	0.300	0.343	0.374	-0.451	-0.568	-0.104	-0.067	-0.026	-0.043
6	f2_2	5	0.294	0.372	0.386	-0.504	-0.431	-0.143	-0.072	-0.054	-0.088
7	f2_1	5	0.295	0.368	0.386	-0.504	-0.443	-0.133	-0.058	-0.063	-0.087
8	p_3	3	0.296	0.353	0.373	-0.461	-0.561	-0.056	-0.073	-0.022	-0.085
9	f_2	5	0.287	0.349	0.366	-0.494	-0.528	0.017	-0.124	-0.128	0.023
10	f_1	5	0.288	0.347	0.369	-0.481	-0.509	0.009	-0.130	-0.136	0.011
11	p_1	3	0.288	0.349	0.366	-0.478	-0.512	0.011	-0.135	0.011	-0.134
12	f2_3	2	0.288	0.359	0.379	-0.468	-0.448	-0.155	-0.013	-0.162	-0.013
13	f2_1	2	0.288	0.358	0.379	-0.441	-0.469	-0.161	-0.013	-0.161	-0.014
14	f2_2	2	0.288	0.358	0.379	-0.439	-0.467	-0.163	-0.013	-0.162	-0.013
15	f_3	3	0.297	0.339	0.358	-0.581	-0.514	0.034	-0.098	-0.098	0.035
16	f_1	3	0.300	0.337	0.356	-0.552	-0.491	0.006	-0.097	-0.097	0.007
17	f2_1	3	0.297	0.357	0.376	-0.497	-0.494	-0.124	0.008	-0.136	-0.015
18	f_2	1	0.300	0.338	0.357	-0.488	-0.515	-0.001	-0.098	-0.126	0.002
19	f2_2	3	0.300	0.355	0.378	-0.482	-0.518	-0.103	-0.018	-0.143	0.005
20	f_2	2	0.296	0.354	0.372	-0.568	-0.528	-0.099	0.029	0.030	-0.099
21	f_1	2	0.296	0.354	0.372	-0.567	-0.527	-0.096	0.026	0.026	-0.096
22	f_3	2	0.296	0.354	0.374	-0.565	-0.526	-0.100	0.028	0.027	-0.100
23	p_1	2	0.296	0.353	0.373	-0.566	-0.528	0.021	-0.092	0.022	-0.092
24	p_2	2	0.295	0.354	0.372	-0.529	-0.491	0.019	-0.126	0.018	-0.126
25	f2_2	1	0.296	0.369	0.388	-0.466	-0.499	-0.017	-0.132	-0.018	-0.132
26	f2_1	1	0.295	0.369	0.387	-0.447	-0.479	-0.026	-0.144	-0.025	-0.142
27	f2_3	1	0.296	0.368	0.387	-0.465	-0.510	-0.019	-0.133	-0.019	-0.118
28	f_1	4	0.309	0.331	0.350	-0.564	-0.525	-0.075	0.017	0.017	-0.075
29	f_3	4	0.308	0.333	0.351	-0.559	-0.521	-0.078	0.014	0.014	-0.078
30	f_2	4	0.308	0.331	0.349	-0.568	-0.530	-0.079	0.027	0.027	-0.079
31	f2_1	4	0.308	0.346	0.365	-0.493	-0.529	-0.005	-0.104	-0.005	-0.099
32	f2_3	4	0.307	0.348	0.364	-0.507	-0.519	-0.007	-0.093	-0.011	-0.098
33	p_3	1	0.309	0.353	0.471	-0.410	-0.488	-0.114	-0.133	-0.000	-0.129
34	f_3	1	0.308	0.355	0.471	-0.432	-0.458	-0.118	-0.111	-0.004	-0.154
35	f_1	1	0.308	0.357	0.471	-0.417	-0.462	-0.118	-0.126	-0.001	-0.154

Table S4: Vibrational frequencies for the structures in group I.

0	1	4	5	0	5	5
p_3	p_1	p_3	p_1	t10_1	f2_2	f2_1
3101.23	3100.28	3101.74	3100.51	3086.87	3086.53	3084.25
3067.38	3067.56	3070.19	3068.23	3044.91	3043.67	3045.12
3020.64	3022.11	3016.68	3030.29	3021.83	3020.88	3019.93
2989.02	2986.86	2989.1	3011.09	2986.64	2984.18	2986.57
1450.54	1448.15	1450.92	1437.75	1452.52	1427.25	1429.27
1432.15	1431.17	1430.74	1425.97	1429.94	1422.63	1423.03
1385.41	1382.57	1386.17	1379.59	1374.49	1387.67	1386.93
1349.59	1347.65	1350.08	1329.74	1336.93	1344.24	1341.55

Continued on next page

1268.5	1265.46	1269.24	1255.29	1244.71	1253.61	1251.89
1211.24	1207.92	1210.37	1200.23	1201.53	1209.32	1208.18
1111.45	1109.15	1109.86	1093.09	1095.41	1085.66	1085.54
1027.76	1026.83	1025.42	1021.66	1024.33	1024.1	1024.52
976.2	975.47	970.06	975.35	970.66	969.22	969.89
871.02	867.49	870.15	857.18	840.86	838.72	836.9
809.65	809.88	786.18	817.72	799.79	818.92	822.43
501.47	497.75	497.24	508.59	502.5	515.87	515.64
390.25	385.99	342.6	406.97	342.48	386.89	386.02
341.8	340.59	217.25	308.02	179.75	229.9	224.14
163.43	160.48	150.08	150.84	156.74	142.31	150.42
134.08	130.11	133.7	122.3	121.42	95.07	98.77
97.12	96.26	101.18	92.37	76.92	77.02	77.45
60.73	62.32	65.79	63.21	61.35	63.3	65.86
44.25	37.35	38.4	28.61	33.49	32.71	36.99
18.29	12.65	24.64	22.82i	29.81	16.57	11.1i
3	5	5	3	2	2	2

p_3	f_2	f_1	p_1	f2_3	f2_1	f2_2
3123.36	3074.43	3074.45	3074.37	3074.89	3074.68	3074.02
3077.48	3065.27	3065.94	3065.82	3065.73	3065.95	3065.53
3043.2	3004.63	3004.5	3004.76	3003.72	3003.69	3003.4
3004.05	2981.23	2980.93	2981.37	2980.64	2980.42	2980.14
1475.74	1442.89	1441.5	1441.81	1440.48	1439.83	1439.56
1444.48	1420.08	1419.5	1419.36	1419.05	1418.48	1417.86
1405.9	1383.83	1383.02	1383.05	1382.92	1382.38	1381.46
1316.83	1337.93	1337.19	1336.96	1336.8	1336.6	1336.36
1270.04	1257.5	1257.34	1257.13	1256.8	1257.48	1257.77
1186.14	1225.02	1222.57	1222.78	1223.52	1222.21	1221.23
1124.42	1063.7	1062.11	1062.34	1063.68	1062.56	1063.43
1097.96	1061.8	1061.99	1061.91	1061.99	1062.36	1061.48
977.37	1010.87	1011.19	1011.19	1015.76	1017.12	1017.24
917.26	895.55	895.04	895.09	904.28	903.66	903.41
823.69	811.95	810.83	810.61	813.1	813.29	812.56
480.52	417.15	418.86	418.89	418.43	419.99	421.04
298.96	404.25	402.62	402.97	404.25	402.49	401.46
260.28	245.68	237.39	232.92	235.15	243.51	236.63
135.67	166.73	163.63	161.01	165.6	165.33	161.74
103.1	141.24	136.47	133.66	139.34	137.7	133.46
91.31	121.11	120.05	120.23	119.22	121.08	122.12
72.58	87.62	85.24	83.51	84.45	87.46	84.9
42.82	55.19	54.5	56.5	55.5	56.22	58.52
26.27	38.6	41.75	40.29	44.02	42.08	42.44
3	3	3	1	3	2	2

f_3	f_1	f2_1	f_2	f2_2	f_2	f_1
3061.62	3063.78	3061.9	3062.98	3062.67	3116.6	3116.78
3057.46	3060.5	3058.84	3058.56	3059.57	3111.43	3111.66
2985.82	2990.98	2988.01	2989.82	2988.15	3012.45	3013.35
2958.76	2964.64	2961.95	2963.55	2961.96	2986.33	2987.2
1413.41	1410.34	1412.19	1409.76	1411.04	1416.57	1415.9
1410.14	1402.74	1408.72	1401.71	1408.38	1412.86	1412.12
1388.56	1387.45	1387.73	1386.76	1386.96	1385.57	1384.86
1345.47	1347.37	1344.47	1346.37	1343.72	1334.77	1333.88
1272.2	1274.46	1271.41	1272.91	1270.73	1248.39	1247.78
1222.18	1224.94	1222.11	1223.72	1221.07	1221.8	1220.99
1094.56	1095.09	1093.01	1094.6	1092.84	1057.43	1057.06
1038.29	1034.12	1040.43	1032.75	1039.52	1051.55	1052.2
1026.82	1022.11	1031.06	1022.4	1030.16	997.39	997.19
881.32	871.48	888.1	871.58	886.98	898.86	897.71
838.74	829.18	840.45	828.87	839.24	796.68	796.68
465.54	467.06	465.27	466.63	463.65	392.62	391.69
390.45	375.06	395.02	376.15	392.16	385.86	386.72
261.39	207.7	266.4	207.93	263.94	136.0	136.04

Continued on next page

143.16	169.8	149.34	167.82	150.49	131.76	130.37
135.23	146.85	140.36	144.52	132.62	114.07	114.98
122.94	119.75	126.15	119.27	117.45	88.12	87.76
104.59	101.13	105.74	102.8	102.38	42.55	49.69
63.94	73.29	66.9	73.36	54.38	38.59	42.28
45.98	47.46	50.81	45.26	39.44	56.29i	53.16i
2	2	2	1	1	1	4

f_3	p_1	p_2	f2_2	f2_1	f2_3	f_1
3115.11	3116.5	-	3114.33	3117.42	3115.01	3040.19
3110.26	3111.24	-	3108.81	3111.33	3109.06	3029.2
3011.77	3012.21	-	3011.52	3014.27	3012.45	2973.54
2985.44	2986.03	-	2985.29	2988.62	2986.35	2948.89
1416.31	1416.17	-	1415.3	1414.28	1415.02	1432.04
1412.48	1412.07	-	1412.11	1410.79	1411.71	1421.51
1384.88	1384.45	-	1383.55	1383.5	1383.31	1368.72
1334.45	1333.88	-	1333.72	1332.66	1333.3	1338.45
1248.99	1247.21	-	1248.67	1245.65	1248.43	1250.13
1220.58	1220.69	-	1219.46	1220.83	1219.35	1206.68
1056.52	1057.38	-	1057.12	1058.6	1056.79	1101.04
1051.86	1051.79	-	1052.36	1052.8	1052.68	1065.28
998.18	996.29	-	1003.91	1001.33	1003.95	1029.54
897.65	897.2	-	906.36	907.46	906.17	891.65
797.59	796.55	-	800.28	797.56	799.56	860.82
390.66	392.44	-	390.66	393.28	390.64	497.72
387.75	386.33	-	388.94	384.52	388.46	350.67
134.77	138.84	-	136.11	135.31	135.95	200.6
133.01	133.79	-	134.26	129.37	135.22	111.62
113.54	117.06	-	113.99	114.1	113.08	94.76
89.48	89.21	-	87.77	86.84	88.37	94.69
53.32	52.06	-	53.12	45.08	51.38	56.59
41.62	46.36	-	42.69	40.65	39.23	47.9
57.26i	53.7i	-	51.76i	53.42i	54.63i	37.26i
4	4	4	4	1	1	1

f_3	f_2	f2_1	f2_3	p_3	f_3	f_1
3039.49	3038.23	3041.36	3043.07	3170.39	3170.2	3171.07
3028.7	3026.79	3030.4	3030.04	3114.49	3116.2	3115.33
2973.53	2971.61	2973.43	2973.32	3072.73	3071.5	3073.44
2948.9	2946.97	2949.07	2948.45	3000.26	2998.78	2998.44
1431.75	1431.59	1429.66	1429.12	1441.49	1441.0	1441.01
1421.81	1421.09	1419.9	1419.09	1429.4	1429.31	1429.19
1368.96	1368.93	1366.6	1367.4	1362.8	1361.16	1361.23
1338.35	1338.49	1337.1	1337.83	1327.49	1313.05	1312.87
1250.16	1249.86	1248.69	1249.66	1227.62	1225.19	1225.24
1206.48	1206.55	1205.28	1206.3	1194.12	1189.67	1189.77
1100.69	1101.81	1099.93	1100.44	1122.61	1117.9	1117.84
1064.93	1065.82	1070.21	1070.82	1018.32	1007.71	1007.77
1029.62	1028.93	1032.99	1032.61	951.81	938.35	938.36
891.63	890.02	894.15	892.92	873.54	871.17	870.78
860.86	859.99	861.88	861.86	726.52	583.16	583.0
497.27	498.09	497.35	497.65	535.41	536.82	537.26
351.33	347.75	349.49	346.51	414.69	486.29	487.02
198.4	197.12	199.09	202.04	283.6	345.69	346.85
111.25	102.95	110.43	107.08	234.11	270.97	271.6
95.21	92.04	97.11	91.22	203.3	233.17	235.01
94.92	91.51	95.36	88.75	167.87	174.96	174.75
58.43	55.86	56.49	59.55	128.69	145.19	146.34
45.35	45.34	46.44	47.48	110.9	125.81	126.71
29.85i	40.26i	36.53i	45.03i	92.24	100.4	103.66

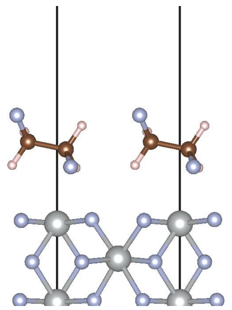

(1) p_3 0

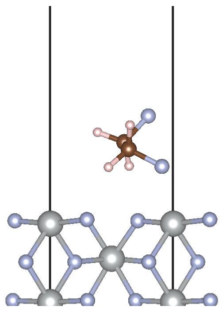

(2) p_1 1

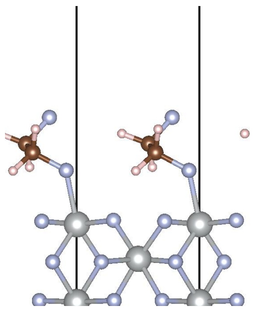

(3) p_3 4

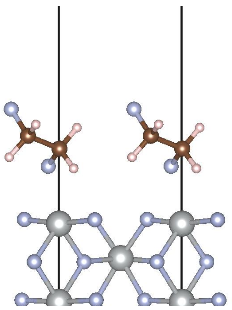

(4) p_1 5

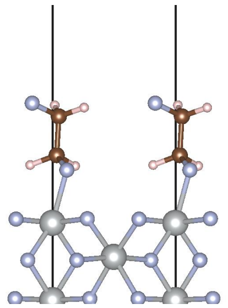

(5) t10_1 0

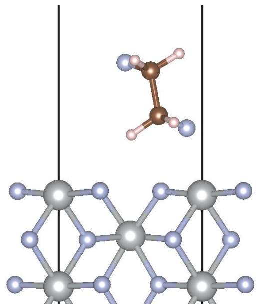

(6) f2_2 5

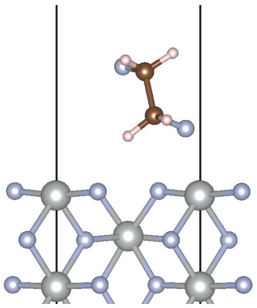

(7) f2_1 5

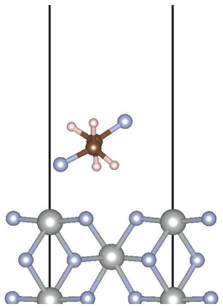

(8) p_3 3

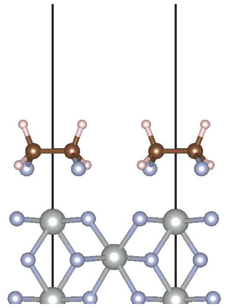

(9) f_2 5

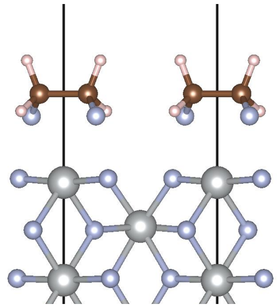

(10) f_1 5

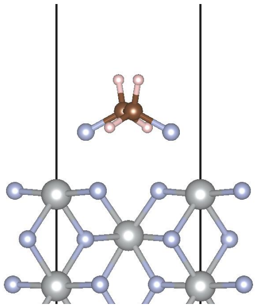

(11) p_1 3

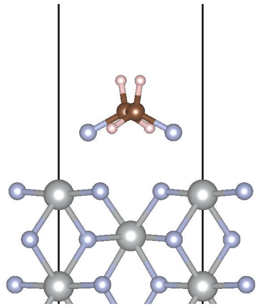

(12) f2_3 2

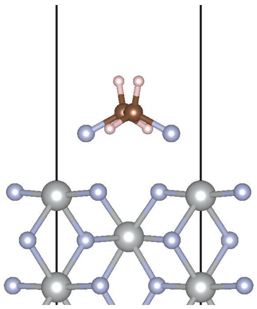

(13) f2_1 2

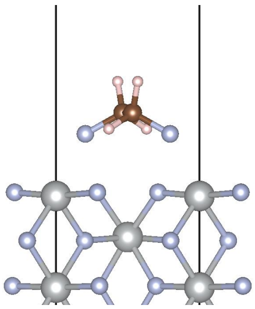

(14) f2_2 2

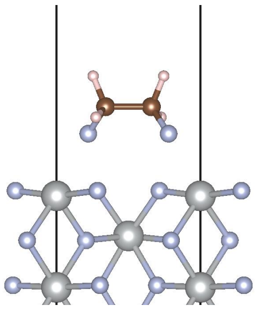

(15) f_3 3

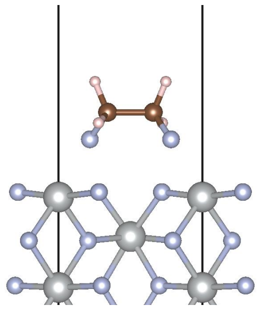

(16) f_1 3

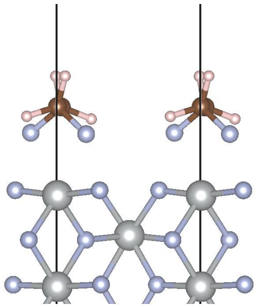

(17) f2_1 3

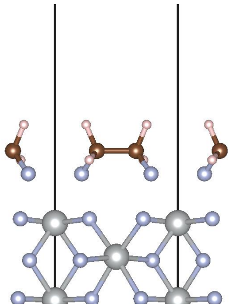

(18) f_2 1

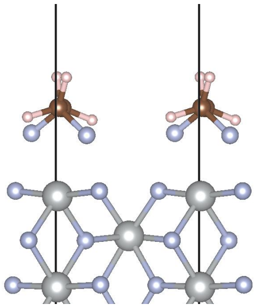

(19) f2_2 3

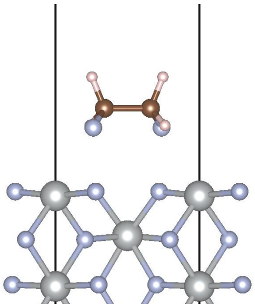

(20) f_2 2

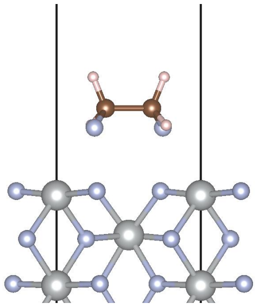

(21) f_1 2

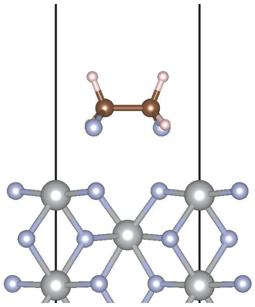

(22) f_3 2

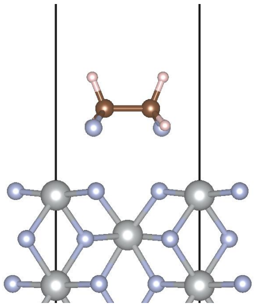

(23) p_1 2

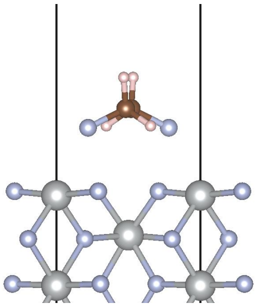

(24) p_2 2

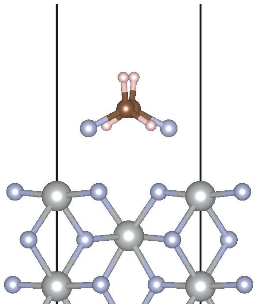

(25) f2_2 1

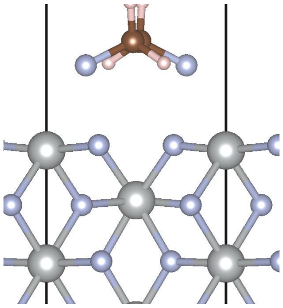

(26) f2_1 1

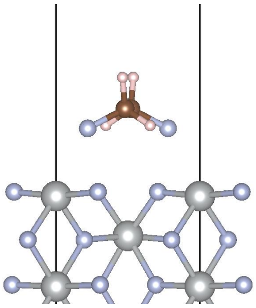

(27) f2_3 1

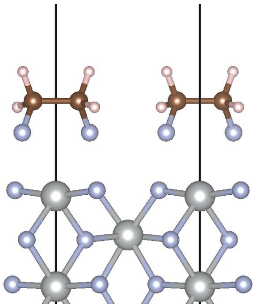

(28) f_1 4

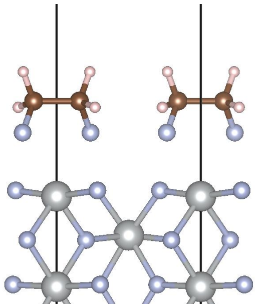

(29) f_3 4

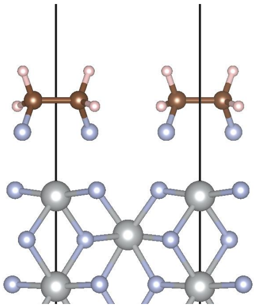

(30) f_2 4

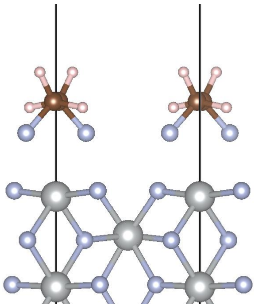

(31) f2_1 4

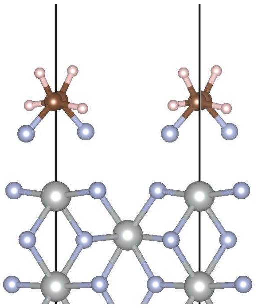

(32) f2_3 4

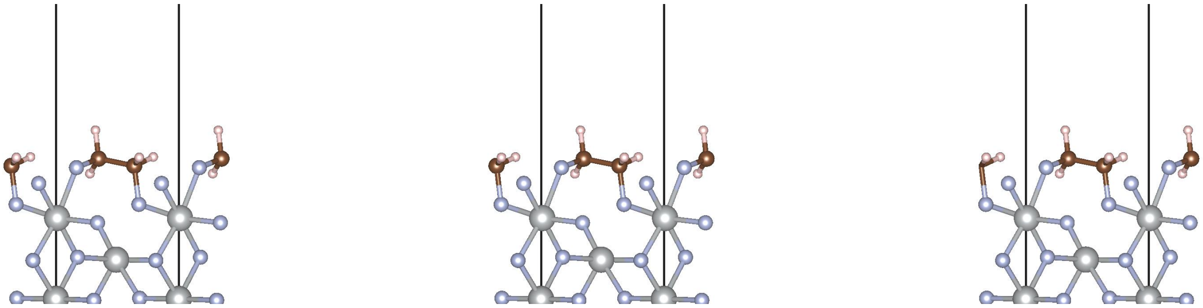

(33) p_3 1 (34) f_3 1 (35) f_1 1

Figure S2: Structures after relaxation in group I.

3 Group II

Table S5: Adsorption energies, and some selected structural parameters for the structures in group II.

#	Orientation	Position	E_{ads}	d(CC)	d(CH)	d(CF)	d(CH)	d(CH)	d(NiF)	d(NiF)	d(HF)
1	f_1	0	-11.1373	1.322	1.087	1.388	1.088	1.088	2.057	2.190	1.047
2	t5_2	2	-11.1142	1.322	1.087	1.389	1.087	1.088	2.058	2.186	1.051
3	f2_1	0	-11.1139	1.322	1.087	1.389	1.088	1.088	2.054	2.192	1.046
4	f_3	5	-11.0897	1.321	1.088	1.391	1.086	1.088	2.040	-	1.040
5	t5_2	1	-11.0897	1.321	1.086	1.391	1.087	1.088	2.040	-	1.039
6	t10_2	1	-11.0895	1.321	1.086	1.391	1.088	1.088	2.039	-	1.040
7	t10_1	1	-11.0894	1.321	1.086	1.391	1.087	1.087	2.040	-	1.040
8	t10_2	5	-11.0893	1.321	1.086	1.391	1.088	1.088	2.040	-	1.039
9	t5_1	1	-11.0889	1.321	1.086	1.391	1.087	1.088	2.040	-	1.040
10	f_2	0	-11.0888	1.321	1.086	1.391	1.087	1.088	2.040	-	1.039
11	p2_1	5	-11.0642	1.321	1.088	1.391	1.087	1.088	2.040	-	1.039
12	p2_1	3	-11.0641	1.321	1.087	1.391	1.088	1.088	2.039	-	1.039
13	t10_1	5	-11.0338	1.322	1.087	1.385	1.087	1.088	2.149	-	1.038
14	t5_1	3	-11.0168	1.322	1.086	1.388	1.087	1.088	2.039	-	1.041
15	t10_2	0	-11.0152	1.322	1.087	1.388	1.087	1.088	2.038	-	1.041
16	t5_1	0	-11.0151	1.322	1.087	1.388	1.087	1.088	2.038	-	1.043
17	p_2	0	-11.0148	1.322	1.087	1.387	1.087	1.088	2.039	-	1.042
18	t10_1	3	-11.0142	1.322	1.087	1.387	1.087	1.088	2.040	-	1.044
19	p_3	2	-10.9970	1.323	1.087	1.382	1.087	1.089	2.048	-	1.049
20	t10_1	4	-10.9758	1.323	1.088	1.377	1.087	1.089	2.057	-	1.054
21	p_3	5	-10.9751	1.323	1.088	1.378	1.088	1.089	2.053	-	1.052
22	t5_2	4	-10.9749	1.323	1.088	1.377	1.088	1.089	2.062	-	1.053
23	p_2	5	-10.9748	1.323	1.088	1.378	1.088	1.089	2.054	-	1.052
24	t10_1	2	-10.9726	1.323	1.088	1.377	1.087	1.089	2.061	-	1.053
25	p2_1	4	-10.9529	1.323	1.088	1.379	1.089	1.090	2.058	-	1.056
26	f2_3	5	-10.9485	1.323	1.089	1.380	1.088	1.090	2.050	-	1.053
27	t5_2	3	-10.9248	1.322	1.087	1.384	1.087	1.088	2.136	-	1.036
28	t10_2	3	-10.9237	1.323	1.088	1.382	1.087	1.088	2.135	-	1.038
29	p_2	1	-10.9111	1.323	1.088	1.378	1.088	1.089	2.146	-	1.041
30	t5_2	0	-10.8888	1.324	1.088	1.375	1.088	1.088	2.161	-	1.042
31	t5_1	2	-10.8866	1.324	1.088	1.376	1.087	1.089	2.159	-	1.042
32	t5_1	5	-9.9875	1.336	1.087	1.359	1.087	1.089	-	-	0.972
33	t5_2	5	-9.9577	1.335	1.087	1.360	1.087	1.089	2.159	-	0.974
34	t10_2	4	-9.8422	1.323	1.087	1.379	1.087	1.088	2.102	-	1.009
35	f_3	0	-9.7648	1.329	1.090	1.385	1.090	1.090	-	-	0.962
36	t5_1	4	-9.7607	1.329	1.089	1.384	1.090	1.090	-	-	0.963
37	t10_2	2	-9.1645	1.318	1.093	1.415	1.088	1.088	1.820	-	1.007

Table S6: Magnetic moments on surface Ni, F and all atoms of ethene for group II.

Ion/Position	Ion	Ion	Ni	F	F	C	C	H	H	H	H
1	f_1	0	-1.828	-0.012	-0.022	-0.000	-0.000	0.000	-0.000	-0.000	-0.000
2	t5_2	2	-1.827	-0.022	-0.012	-0.000	-0.001	0.000	-0.000	-0.000	-0.000
3	f2_1	0	-1.827	-0.012	-0.022	-0.001	-0.000	-0.000	0.000	-0.000	-0.000
4	f_3	5	-1.828	-0.023	-0.008	-0.001	-0.000	-0.000	-0.000	-0.000	0.000
5	t5_2	1	-1.828	-0.008	-0.023	-0.000	-0.001	0.000	-0.000	-0.000	-0.000
6	t10_2	1	-1.828	-0.008	-0.023	-0.000	-0.001	-0.000	0.000	-0.000	-0.000
7	t10_1	1	-1.828	-0.008	-0.023	-0.000	-0.001	-0.000	0.000	-0.000	-0.000
8	t10_2	5	-1.828	-0.008	-0.023	-0.000	-0.001	-0.000	0.000	-0.000	-0.000
9	t5_1	1	-1.828	-0.008	-0.023	-0.000	-0.001	-0.000	0.000	-0.000	-0.000
10	f_2	0	-1.828	-0.008	-0.023	-0.000	-0.001	0.000	-0.000	-0.000	-0.000
11	p2_1	5	-1.828	-0.008	-0.023	-0.001	-0.001	-0.000	0.000	-0.000	-0.000
12	p2_1	3	-1.828	-0.008	-0.023	-0.001	-0.001	0.000	-0.000	-0.000	-0.000
13	t10_1	5	-1.831	-0.008	-0.021	-0.000	-0.000	-0.000	0.000	-0.000	-0.000
14	t5_1	3	-1.828	-0.009	-0.023	-0.001	-0.001	0.000	-0.000	-0.000	-0.000
15	t10_2	0	-1.828	-0.009	-0.023	-0.000	-0.001	0.000	-0.000	-0.000	-0.000

Continued on next page

Ion/Position	Ion	Ion	Ni	F	F	C	C	H	H	H	H
16	t5_1	0	-1.828	-0.009	-0.023	-0.000	-0.001	-0.000	-0.000	-0.000	-0.000
17	p_2	0	-1.828	-0.009	-0.023	-0.000	-0.001	-0.000	-0.000	-0.000	-0.000
18	t10_1	3	-1.827	-0.009	-0.023	-0.000	-0.001	-0.000	-0.000	-0.000	-0.000
19	p_3	2	-1.827	-0.023	-0.009	-0.001	-0.000	-0.000	-0.000	-0.000	-0.000
20	t10_1	4	-1.827	-0.022	-0.009	-0.001	-0.000	0.000	-0.000	-0.000	-0.000
21	p_3	5	-1.827	-0.009	-0.023	-0.001	-0.001	-0.000	-0.000	0.000	-0.000
22	t5_2	4	-1.827	-0.022	-0.010	-0.001	-0.000	0.000	-0.000	-0.000	-0.000
23	p_2	5	-1.827	-0.023	-0.009	-0.001	-0.001	0.000	-0.000	-0.000	-0.000
24	t10_1	2	-1.827	-0.010	-0.022	-0.001	-0.000	-0.000	0.000	-0.000	-0.000
25	p2_1	4	-1.826	-0.022	-0.008	-0.002	-0.000	-0.000	-0.000	-0.000	-0.000
26	f2_3	5	-1.827	-0.023	-0.009	-0.001	-0.001	-0.000	-0.000	0.000	-0.000
27	t5_2	3	-1.831	-0.007	-0.021	-0.000	-0.001	0.000	-0.000	-0.000	-0.000
28	t10_2	3	-1.830	-0.007	-0.021	0.000	-0.001	-0.000	-0.000	-0.000	-0.000
29	p_2	1	-1.830	-0.006	-0.020	-0.000	-0.001	-0.000	-0.000	-0.000	-0.000
30	t5_2	0	-1.830	-0.007	-0.020	-0.000	-0.000	-0.000	0.000	-0.000	-0.000
31	t5_1	2	-1.830	-0.007	-0.020	-0.000	-0.000	-0.000	0.000	-0.000	-0.000
32	t5_1	5	-1.821	-0.003	-0.010	-0.000	0.000	-0.000	-0.000	-0.000	-0.000
33	t5_2	5	-1.819	-0.003	-0.012	-0.001	0.000	-0.000	-0.000	-0.000	-0.000
34	t10_2	4	-1.818	-0.017	-0.006	-0.000	-0.001	-0.000	0.001	-0.000	-0.000
35	f_3	0	-1.815	-0.008	-0.000	-0.000	-0.000	0.000	-0.000	0.000	0.000
36	t5_1	4	-1.815	-0.000	-0.008	-0.000	-0.000	0.000	0.000	0.000	-0.000
37	t10_2	2	-1.817	-0.063	-0.000	-0.000	0.000	0.000	-0.000	-0.000	-0.000

Table S7: Charge transfer on surface Ni, F and all atoms of ethene for group II.

Ion/Position	Orientation	Position	Ni	F	F	C	C	H	H	H	H
1	f_1	0	0.283	0.359	0.490	-0.461	0.006	-0.143	-0.699	-0.037	-0.045
2	t5_2	2	0.283	0.469	0.370	-0.468	0.001	-0.139	-0.696	-0.014	-0.053
3	f2_1	0	0.283	0.372	0.489	-0.266	-0.137	-0.049	-0.167	-0.698	-0.075
4	f_3	5	0.276	0.465	0.386	-0.017	-0.450	-0.039	-0.040	-0.696	-0.141
5	t5_2	1	0.277	0.367	0.483	-0.452	-0.018	-0.136	-0.694	-0.039	-0.042
6	t10_2	1	0.277	0.367	0.484	-0.451	-0.015	-0.696	-0.141	-0.041	-0.040
7	t10_1	1	0.275	0.367	0.483	-0.457	-0.015	-0.695	-0.138	-0.039	-0.038
8	t10_2	5	0.273	0.368	0.488	-0.438	-0.034	-0.699	-0.142	-0.034	-0.040
9	t5_1	1	0.276	0.369	0.483	-0.457	-0.019	-0.695	-0.137	-0.043	-0.032
10	f_2	0	0.274	0.369	0.489	-0.458	-0.015	-0.139	-0.701	-0.033	-0.044
11	p2_1	5	0.278	0.382	0.482	-0.086	-0.328	-0.068	-0.164	-0.694	-0.055
12	p2_1	3	0.282	0.379	0.487	-0.351	-0.060	-0.160	-0.064	-0.063	-0.700
13	t10_1	5	0.275	0.364	0.497	-0.458	0.005	-0.702	-0.151	-0.048	-0.029
14	t5_1	3	0.274	0.364	0.487	-0.443	-0.006	-0.159	-0.700	-0.035	-0.038
15	t10_2	0	0.276	0.364	0.484	-0.487	-0.002	-0.135	-0.695	-0.026	-0.032
16	t5_1	0	0.277	0.360	0.483	-0.462	0.009	-0.146	-0.693	-0.047	-0.035
17	p_2	0	0.276	0.356	0.486	-0.476	0.008	-0.135	-0.696	-0.039	-0.034
18	t10_1	3	0.278	0.368	0.493	-0.490	-0.013	-0.122	-0.702	-0.035	-0.027
19	p_3	2	0.279	0.477	0.377	0.019	-0.512	-0.041	-0.060	-0.087	-0.701
20	t10_1	4	0.278	0.475	0.374	-0.484	-0.015	-0.079	-0.698	-0.085	-0.014
21	p_3	5	0.283	0.353	0.496	-0.027	-0.480	-0.007	-0.081	-0.080	-0.701
22	t5_2	4	0.282	0.474	0.369	-0.505	-0.009	-0.076	-0.697	-0.068	-0.013
23	p_2	5	0.277	0.477	0.371	-0.503	-0.007	-0.082	-0.699	-0.017	-0.066
24	t10_1	2	0.287	0.350	0.496	-0.499	0.012	-0.701	-0.086	-0.074	-0.025
25	p2_1	4	0.279	0.477	0.389	-0.119	-0.303	-0.031	-0.102	-0.701	-0.133
26	f2_3	5	0.282	0.477	0.383	-0.308	-0.109	-0.108	-0.700	-0.106	-0.055
27	t5_2	3	0.267	0.360	0.493	-0.485	-0.008	-0.120	-0.699	-0.026	-0.036
28	t10_2	3	0.274	0.367	0.499	-0.507	0.011	-0.103	-0.702	-0.019	-0.066
29	p_2	1	0.272	0.359	0.498	-0.501	0.005	-0.087	-0.700	-0.027	-0.064
30	t5_2	0	0.269	0.351	0.497	-0.494	0.010	-0.699	-0.099	-0.052	-0.030
31	t5_1	2	0.270	0.352	0.498	-0.481	0.012	-0.700	-0.106	-0.065	-0.026
32	t5_1	5	0.287	0.370	0.489	-0.513	0.078	-0.202	-0.670	-0.061	-0.025
33	t5_2	5	0.292	0.371	0.461	-0.500	0.067	-0.198	-0.651	-0.039	-0.051
34	t10_2	4	0.298	0.465	0.374	-0.509	-0.002	-0.089	-0.695	-0.036	-0.028

Continued on next page

Ion/Position	Orientation	Position	Ni	F	F	C	C	H	H	H	H
35	f_3	0	0.299	0.353	0.491	0.048	-0.446	-0.083	-0.103	-0.643	-0.146
36	t5_1	4	0.299	0.480	0.375	-0.459	0.026	-0.651	-0.147	-0.075	-0.079
37	t10_2	2	0.317	0.446	0.377	-0.409	-0.020	-0.722	-0.181	-0.047	-0.034

Table S8: Vibrational frequencies for the structures in group I.

0	2	0	5	1	1	1
f_1	t5_2	f2_1	f_3	t5_2	t10_2	t10_1
3206.27	3207.61	3209.09	3210.83	3211.65	3209.32	3212.3
3181.54	3175.35	3178.33	3190.83	3190.24	3189.05	3191.77
3108.92	3110.35	3110.32	3114.37	3115.46	3113.04	3116.16
2135.47	2090.67	2146.7	2219.73	2225.44	2222.3	2216.58
1657.72	1660.51	1657.9	1657.5	1657.93	1656.92	1657.58
1351.97	1351.77	1351.7	1351.32	1351.44	1351.7	1351.34
1293.39	1295.79	1292.67	1292.8	1293.25	1292.48	1292.78
1191.69	1201.49	1187.51	1178.53	1177.33	1178.25	1178.4
1079.1	1079.72	1078.53	1066.84	1066.28	1066.45	1066.36
1035.14	1040.59	1031.57	1038.52	1036.01	1037.77	1038.15
941.83	942.26	946.09	937.72	935.05	935.45	934.87
878.06	876.45	879.51	868.26	868.05	868.5	868.1
839.52	830.36	846.28	847.5	847.34	848.76	847.91
707.5	708.21	707.9	700.03	699.36	700.46	699.07
482.83	476.96	482.5	471.47	471.34	472.11	471.07
369.3	370.29	368.61	366.51	366.51	366.97	367.89
253.78	251.87	256.07	258.8	258.9	258.57	259.6
185.12	184.21	186.85	166.35	165.18	166.14	163.79
150.99	162.47	156.78	143.81	137.05	140.71	137.94
132.84	117.59	141.8	105.37	104.87	109.3	108.32
112.36	91.86	111.41	102.78	101.74	102.14	101.98
77.77	88.96	79.52	81.21	81.25	83.88	82.75
53.12	57.67	58.0	62.89	64.05	67.88	64.2
28.57	22.36i	36.31	36.3	35.48	43.37	46.71
5	1	0	5	3	5	3
t10_2	t5_1	f_2	p2_1	p2_1	t10_1	t5_1
3211.93	3212.27	3211.5	3212.26	3211.5	3207.9	3210.78
3190.13	3191.19	3191.34	3188.36	3186.39	3175.23	3187.59
3115.98	3116.19	3115.06	3114.03	3113.2	3111.27	3114.1
2223.47	2216.64	2225.97	2223.5	2232.69	2247.04	2202.11
1656.91	1657.52	1657.64	1657.97	1658.89	1656.92	1661.97
1351.39	1351.58	1351.99	1350.59	1350.37	1354.2	1351.44
1293.83	1292.11	1292.6	1292.45	1290.95	1297.19	1286.26
1177.13	1178.84	1177.33	1177.61	1175.75	1170.26	1187.96
1065.91	1066.12	1066.37	1068.82	1069.62	1080.52	1070.1
1037.73	1036.59	1036.31	1037.15	1038.83	1011.58	1042.64
937.7	931.9	931.44	937.37	939.47	946.78	935.57
867.91	868.1	868.64	871.19	871.79	898.08	874.49
848.24	849.26	848.83	849.22	846.96	845.71	832.57
700.55	699.58	698.8	699.72	702.67	707.51	700.21
470.95	471.69	472.16	470.71	471.82	627.05	468.82
366.7	365.65	366.57	366.48	365.8	450.08	357.31
259.57	258.57	258.38	259.65	259.32	323.13	259.8
165.54	164.87	166.0	165.66	169.37	181.43	177.23
145.38	131.9	131.79	142.74	143.63	154.54	141.97
109.71	106.32	109.8	107.13	111.11	125.32	121.47
101.44	101.81	102.45	104.09	99.87	98.47	101.32
85.6	82.2	81.57	82.38	84.52	61.2	90.08
64.37	63.94	60.93	64.9	69.26	51.82	58.83
45.91	53.19	52.04	47.33	37.44	26.12	25.77i
0	0	0	3	2	4	5

Continued on next page

t10_2	t5_1	p_2	t10_1	p_3	t10_1	p_3
3209.32	3209.46	3209.18	3208.63	3208.68	3204.5	3203.77
3185.96	3183.72	3182.59	3181.19	3177.79	3163.91	3161.47
3112.85	3113.28	3112.98	3112.48	3109.83	3102.86	3101.96
2196.75	2176.25	2182.74	2164.96	2101.49	2047.17	2070.41
1661.31	1659.4	1659.55	1658.56	1651.64	1655.81	1655.74
1351.53	1352.0	1352.06	1353.04	1348.28	1352.89	1354.34
1289.6	1288.69	1288.98	1290.61	1284.05	1282.68	1283.06
1188.95	1191.41	1190.58	1193.54	1202.58	1222.44	1217.43
1070.36	1072.78	1073.49	1076.75	1096.0	1109.26	1105.37
1039.16	1039.01	1038.55	1038.33	1031.9	1029.83	1022.98
934.89	929.97	930.39	928.64	927.02	920.59	921.77
874.11	874.07	875.09	876.19	880.58	893.76	892.29
834.84	837.51	837.41	840.36	853.71	856.33	858.76
703.22	704.75	704.5	704.98	708.79	696.32	699.87
466.35	463.37	463.74	464.11	468.21	484.31	484.22
355.29	354.64	354.5	352.97	360.07	362.99	361.45
257.14	256.67	259.06	260.4	251.98	253.15	253.0
179.14	176.16	176.18	175.14	175.52	167.6	162.09
148.73	149.45	148.45	155.85	163.57	142.64	146.3
120.65	112.26	112.21	110.03	121.55	109.47	100.21
97.07	96.02	96.31	99.19	92.09	88.91	91.33
88.36	79.08	79.39	75.79	77.06	84.67	81.19
50.95	49.67	46.6	57.34	47.77	47.25	52.7
15.43	24.81	27.76	13.73	44.79	17.06i	31.58
4	5	2	4	5	3	3
t5_2	p_2	t10_1	p2_1	f2_3	t5_2	t10_2
3205.15	3204.13	3206.3	3202.71	3203.82	3211.22	3208.1
3167.5	3164.0	3167.0	3158.24	3160.47	3183.81	3172.08
3105.42	3103.15	3106.4	3096.22	3099.75	3115.26	3111.28
2053.06	2069.52	2058.0	2033.06	2061.1	2275.6	2241.45
1656.74	1656.25	1656.98	1655.08	1655.57	1661.45	1655.72
1351.66	1352.79	1351.18	1348.86	1353.35	1353.53	1353.25
1282.48	1283.21	1282.07	1288.59	1282.36	1290.83	1291.29
1219.07	1219.34	1218.56	1223.75	1218.47	1180.78	1183.26
1112.4	1105.53	1111.08	1111.73	1104.37	1080.54	1088.9
1024.41	1023.3	1023.24	1031.51	1026.48	1018.81	1015.58
922.17	923.34	921.37	920.02	921.24	928.28	927.14
894.34	892.08	893.97	890.25	891.23	892.67	889.85
847.1	856.4	847.16	870.45	860.8	833.58	842.3
698.69	698.58	698.81	703.37	697.72	703.31	708.84
482.43	483.5	482.11	466.24	481.44	590.79	587.99
362.29	361.02	363.81	367.25	361.03	454.39	461.3
242.59	251.77	242.87	255.36	258.58	321.75	313.17
160.11	163.71	161.21	166.47	166.95	169.23	167.06
141.29	142.94	143.66	161.38	142.43	133.49	141.85
104.76	110.49	104.05	102.17	96.77	108.2	108.06
90.21	93.81	91.15	84.18	87.13	78.58	80.82
84.33	82.82	85.33	69.28	77.49	66.25	68.44
32.09	45.38	30.76	56.94	44.51	47.15	44.04
22.12i	27.95	17.99i	22.75	35.61	14.51i	18.83
1	0	2	5	5	4	0
p_2	t5_2	t5_1	t5_1	t5_2	t10_2	f_3
3206.16	3206.35	3206.73	3274.89	3244.56	3206.12	3447.6
3167.78	3166.14	3165.81	3207.63	3209.91	3173.73	3190.34
3107.32	3108.11	3108.13	3182.13	3180.33	3109.5	3146.95
2205.77	2193.64	2198.78	3103.47	3104.47	2697.92	3087.84
1651.6	1657.98	1657.64	1620.84	1621.15	1661.66	1634.09
1350.44	1353.18	1353.48	1348.75	1350.14	1352.53	1356.88
1284.58	1286.0	1286.23	1279.29	1277.43	1287.43	1276.44
1189.78	1194.16	1194.78	1126.3	1125.75	1102.89	1088.15

Continued on next page

1108.64	1114.67	1113.28	973.7	979.19	1094.53	985.47
1012.08	999.04	998.98	915.29	914.27	927.34	917.19
924.12	926.4	925.52	859.62	859.67	886.55	889.63
893.68	905.07	904.58	801.36	814.91	835.08	804.02
852.63	838.21	839.59	705.57	713.78	826.28	613.83
710.11	702.04	702.3	585.68	582.04	702.22	551.34
584.4	605.77	605.71	475.86	473.34	470.19	518.8
470.09	452.56	452.98	407.11	256.24	264.96	244.85
326.7	328.38	326.54	246.1	225.19	223.76	201.29
155.88	144.25	147.39	185.32	198.86	154.0	176.63
146.52	127.29	126.06	158.36	155.62	132.07	131.91
111.76	102.05	99.62	128.22	127.46	115.95	103.76
76.47	79.28	79.56	85.03	79.23	86.19	86.22
63.25	62.22	70.18	68.36	70.06	52.95	61.38
39.95	32.33	34.33	58.1	49.79	50.71	48.41
24.5	9.92	20.7	37.46	21.37	26.34i	25.19
4	2					

t5_1	t10_2
3442.09	3207.58
3190.19	3114.41
3152.39	3087.83
3087.71	2698.69
1633.39	1663.69
1356.39	1355.39
1275.39	1306.65
1089.91	1045.31
982.67	957.36
918.45	950.47
891.98	877.9
804.41	848.21
615.28	831.15
553.97	702.96
518.84	490.59
242.87	476.51
196.78	189.95
179.44	149.31
127.82	137.85
97.13	108.95
77.59	92.43
43.78	77.59
30.11	40.26
21.86i	29.53

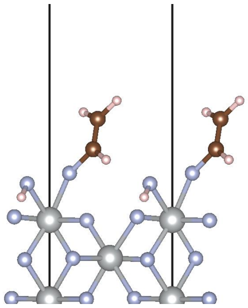
(1) f_1 0

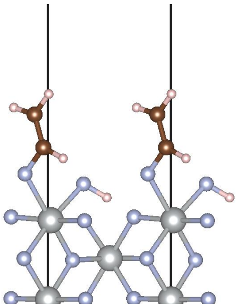
(2) t5_2 2

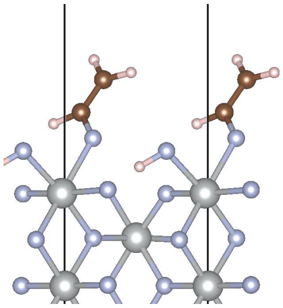
(3) f2_1 0

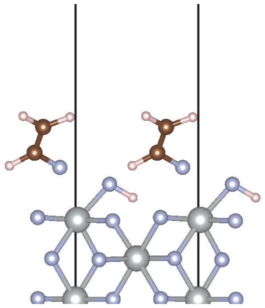
(4) f_3 5

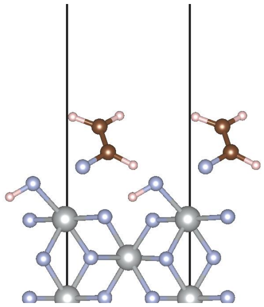
(5) t5_2 1

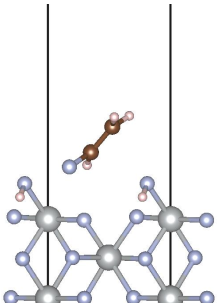
(6) t10_2 1

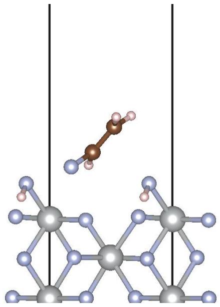
(7) t10_1 1

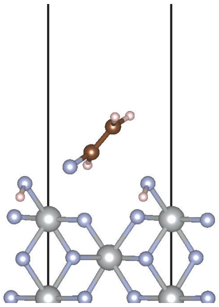
(8) t10_2 5

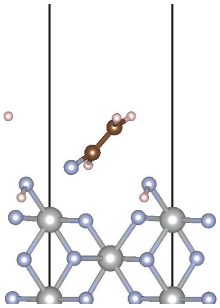
(9) t5_1 1

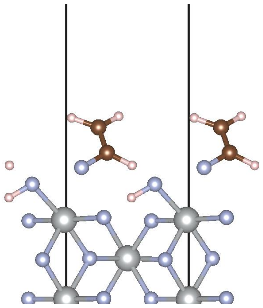
(10) f_2 0

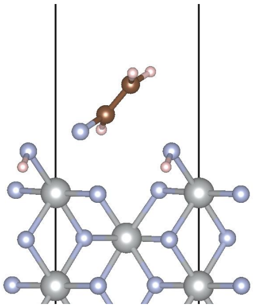
(11) p2_1 5

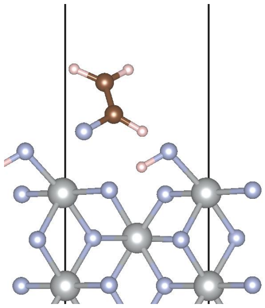
(12) p2_1 3

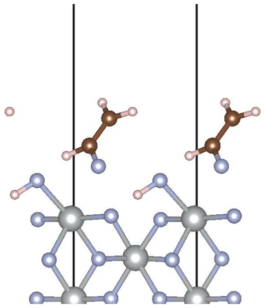
(13) t10_1 5

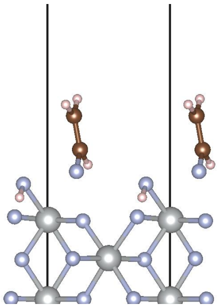
(14) t5_1 3

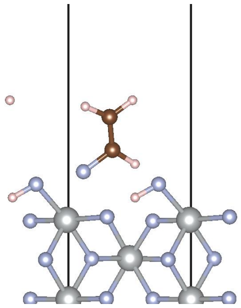
(15) t10_2 0

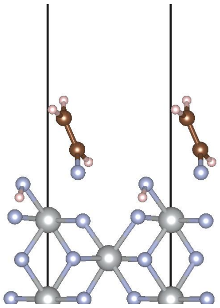
(16) t5_1 0

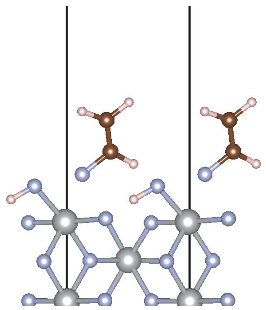
(17) p_2 0

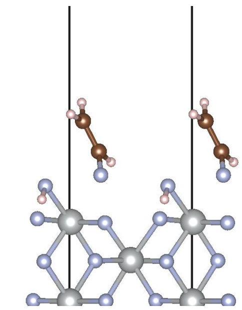
(18) t10_1 3

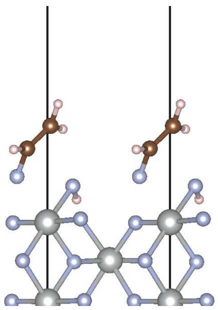
(19) p_3 2

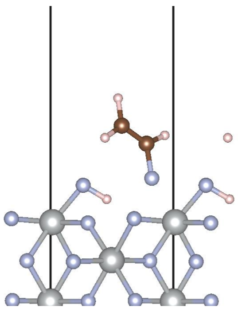
(20) t10_1 4

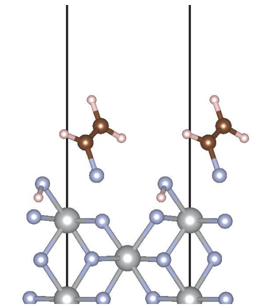
(21) p_3 5

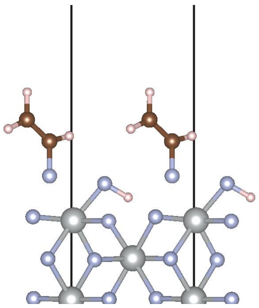
(22) t5_2 4

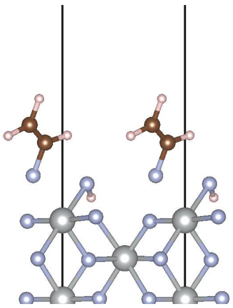
(23) p_2 5

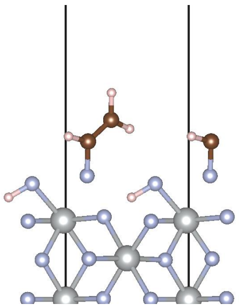
(24) t10_1 2

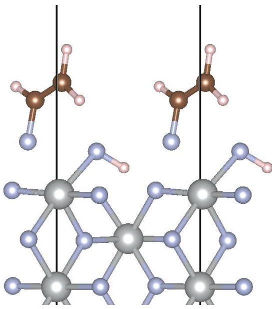
(25) p2_1 4

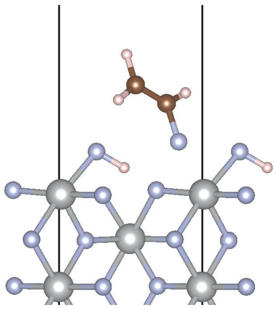
(26) f2_3 5

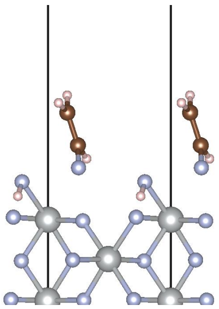
(27) t5_2 3

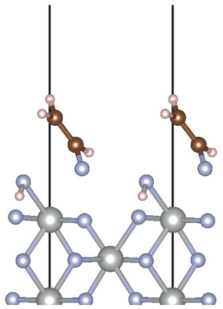
(28) t10_2 3

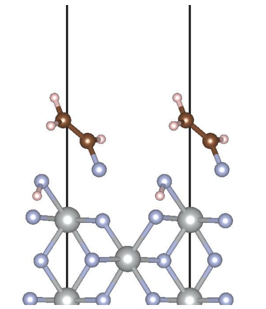
(29) p_2 1

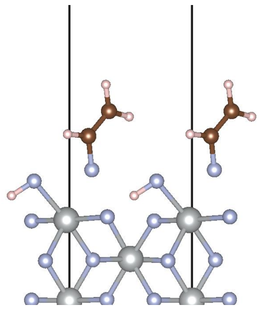
(30) t5_2 0

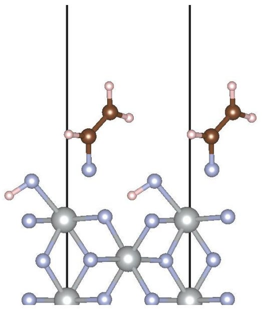
(31) t5_1 2

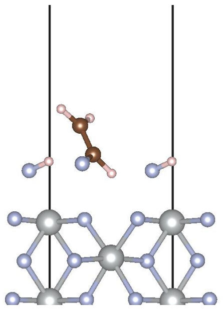
(32) t5_1 5

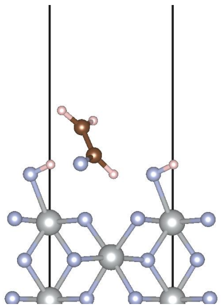

(33) t5_2 5

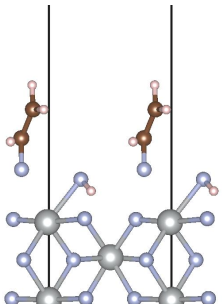

(34) t10_2 4

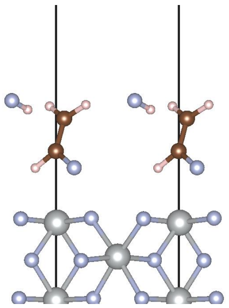

(35) f_3 0

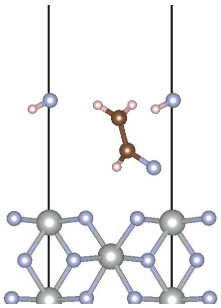

(36) t5_1 4

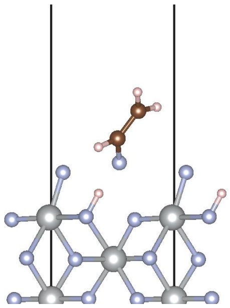

(37) t10_2 2

Figure S3: Structures after relaxation in group II.

4 Group III

Table S9: Adsorption energies, and some selected structural parameters for the structures in group III.

#	Orientation	Position	E_{ads}	d(CC)	d(CH)	d(CH/F)	d(NiF)	d(NiF)	d(HF)	d(HF/H)
1	p2_1	0	-9.181	1.208	1.071	1.071	2.038	2.037	0.992	0.991
2	p2_2	1	-3.757	1.208	1.068	1.275	2.149	-	0.968	0.751

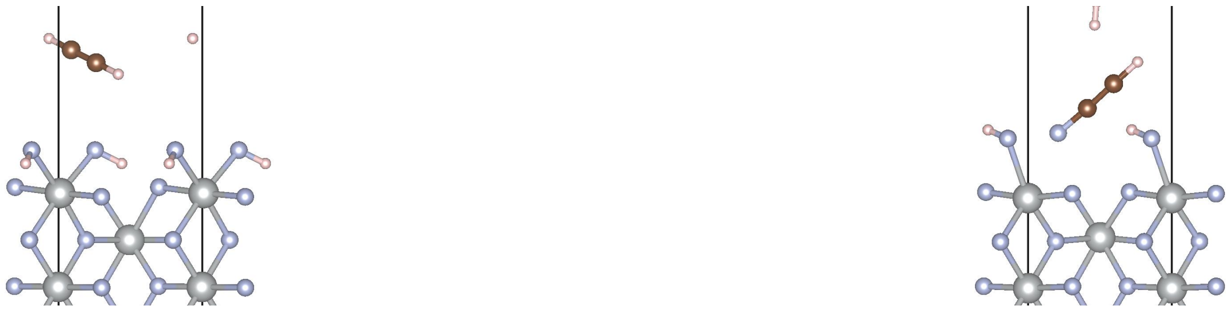

(1) p2_1 0

(2) p2_2 1

Figure S4: Structures after relaxation in group III.

Table S10: Magnetic moments on surface Ni, F and all atoms of ethene for group III.

Ion/Position	Ion	Ion	Ni	F	F	C	C	H	H	H	H
1	p2_1	0	-1.831	-0.022	-0.022	-0.000	-0.000	-0.000	0.000	-0.000	-0.000
2	p2_2	1	-1.816	-0.001	-0.012	-0.000	0.000	0.000	0.000	-0.000	0.000

Table S11: Charge transfer on surface Ni, F and all atoms of ethene for group III.

Ion/Position	Orientation	Position	Ni	F	F	C	C	H	H	H	H
1	p2_1	0	0.281	0.464	0.482	-0.011	0.305	-0.177	-0.182	-0.718	-0.717
2	p2_2	1	0.304	0.343	0.474	-0.198	-0.365	0.040	0.020	-0.182	-0.672

Table S12: Vibrational frequencies for the structures in group III.

0	1
p2_1	p2_2
3448.61	4291.69
3349.89	3422.93
3031.34	3392.1
2829.64	2248.13
2004.56	1086.32
1113.06	684.76
973.07	626.57
831.38	531.38
754.38	506.33
745.06	373.69
712.75	352.35
618.29	236.55
605.59	226.16
334.12	183.46
294.09	160.24

Continued on next page

236.77	145.54
199.33	129.18
139.02	117.05
131.4	101.1
67.31	82.04
41.19	51.11
23.04	35.11
9.92	25.84
11.84i	54.81i

5 Group IV

Table S13: Adsorption energies, and some selected structural parameters for the structures in group IV.

#	Orientation	Position	E_{ads}	d(CC)	d(CH)	d(CF)	d(CH)	d(CF)	d(HH)	d(NiF)
1	p2_1	1	-6.9564	1.327	1.088	1.358	1.089	1.385	0.751	-
2	p2_2	2	-6.8021	1.326	1.088	1.364	1.090	1.388	0.751	-
3	p2_1	2	-4.3907	1.313	1.085	1.363	1.075	1.397	0.755	1.817
4	p2_2	5	-4.3608	1.315	1.086	1.361	1.076	1.394	0.751	1.804

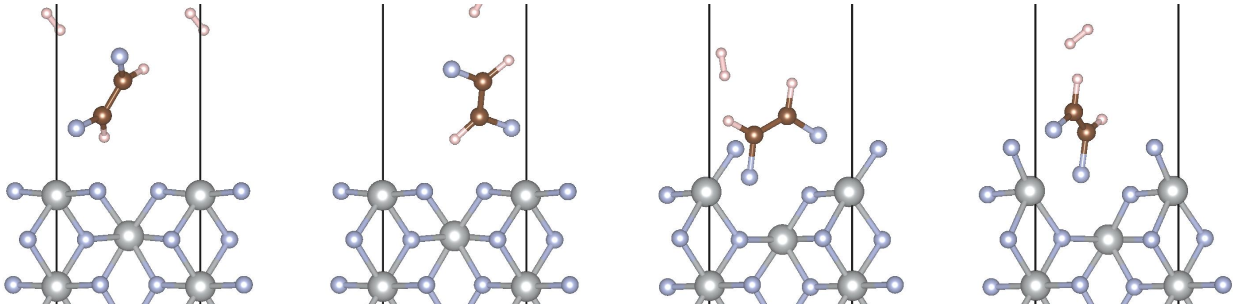

(1) p2_1 1 (2) p2_2 2 (3) p2_1 2 (4) p2_2 5

Figure S5: Structures after relaxation in group IV.

Table S14: Magnetic moments on surface Ni, F and all atoms of ethene for group IV.

Ion/Position	Ion	Ion	Ni	F	F	C	C	H	H	H	H
1	p2_1	1	-1.816	-0.008	-0.000	-0.000	-0.000	-0.000	-0.000	-0.000	-0.000
2	p2_2	2	-1.816	-0.000	-0.008	-0.000	-0.000	-0.000	-0.000	-0.000	-0.000
3	p2_1	2	-1.809	-0.057	-0.007	-0.005	0.002	-0.000	-0.000	-0.000	-0.000
4	p2_2	5	-1.807	-0.006	-0.060	-0.004	0.001	0.000	0.000	-0.000	-0.000

Table S15: Charge transfer on surface Ni, F and all atoms of ethene for group IV.

Ion/Position	Orientation	Position	Ni	F	F	C	C	H	H	H	H
1	p2_1	1	0.291	0.377	0.384	-0.556	-0.481	0.026	0.026	-0.187	-0.116
2	p2_2	2	0.291	0.367	0.392	-0.575	-0.468	0.039	0.015	-0.190	-0.106
3	p2_1	2	0.312	0.428	0.398	-0.600	-0.510	-0.026	0.085	-0.165	-0.091
4	p2_2	5	0.315	0.379	0.438	-0.633	-0.504	0.032	0.022	-0.161	-0.062

Table S16: Vibrational frequencies for the structures in group IV.

1	2	2	5
p2_1	p2_2	p2_1	p2_2
4282.44	4291.62	4202.78	4278.85
3170.14	3163.51	3298.58	3284.85
3143.56	3132.68	3192.68	3191.24
1699.94	1686.33	1742.6	1738.44
1329.05	1285.61	1290.35	1297.53
1232.63	1251.39	1207.91	1215.4
1072.82	1106.66	1016.16	1028.42
946.54	1041.32	809.78	813.39
854.09	896.04	795.53	795.89
767.96	789.27	689.0	692.12
749.27	562.26	585.34	591.05
489.6	352.32	507.24	520.59
320.55	317.12	452.12	454.79
210.73	208.69	417.8	311.93
148.21	187.82	362.96	254.81
133.83	172.57	307.91	193.23
115.71	149.68	196.83	180.8
101.58	141.43	179.03	176.35
83.1	99.11	173.01	145.52
77.42	84.03	140.31	126.51
62.08	60.49	138.41	118.65
30.92	49.52	107.97	107.56
4.02	25.88	93.14	74.13
27.53i	63.35i	70.4	61.09i

6 Group V

Table S17: Adsorption energies, and some selected structural parameters for the structures in group V.

#	Orientation	Position	E_{ads}	d(CH)	d(CH)	d(CF)	d(CF)	d(CH)	d(CH)	d(NiF)
1	pv	1	0.7183	1.091	1.094	1.371	1.467	1.120	1.120	1.841
2	pv	3	0.7198	1.091	1.094	1.371	1.468	1.120	1.120	1.841
3	pv	0	0.7285	1.091	1.094	1.371	1.468	1.121	1.121	1.841
4	pv	2	0.7285	1.091	1.094	1.371	1.467	1.119	1.120	1.841
5	pv	4	2.0324	1.087	1.091	1.397	1.419	1.120	1.120	1.852

Table S18: Magnetic moments on surface Ni, F and all atoms of ethene for group V.

Ion/Position	Ion	Ion	Ni	F	F	C	C	H	H	H	H
1	pv	1	-1.819	-0.051	-0.007	-0.000	-0.000	-0.001	0.000	0.000	0.000
2	pv	3	-1.819	-0.007	-0.051	-0.000	-0.000	0.000	-0.001	0.000	-0.000
3	pv	0	-1.819	-0.051	-0.007	-0.000	-0.000	-0.001	0.000	-0.000	-0.000
4	pv	2	-1.819	-0.051	-0.007	0.000	-0.000	0.000	-0.001	0.000	0.000
5	pv	4	-1.816	-0.011	-0.059	0.000	0.000	-0.000	0.000	0.000	0.000

Table S19: Charge transfer on surface Ni, F and all atoms of ethene for group V.

Ion/Position	Orientation	Position	Ni	F	F	C	C	H	H	H	H
1	pv	1	0.304	0.447	0.380	0.006	-0.921	-0.195	-0.159	0.001	-0.007
2	pv	3	0.304	0.361	0.465	0.020	-0.923	-0.160	-0.191	-0.012	-0.008

Continued on next page

Ion/Position	Orientation	Position	Ni	F	F	C	C	H	H	H	H
3	pv	0	0.305	0.447	0.382	0.060	-0.935	-0.182	-0.160	-0.027	-0.033
4	pv	2	0.305	0.447	0.383	0.024	-0.937	-0.143	-0.198	-0.003	-0.021
5	pv	4	0.314	0.344	0.458	0.019	-1.020	-0.119	-0.111	-0.011	-0.007

Table S20: Vibrational frequencies for the structures in group V.

1	3	0	2	4
pv	pv	pv	pv	pv
3157.63	3158.06	3158.4	3156.69	3207.02
3046.34	3046.73	3048.03	3045.25	3089.74
2912.58	2910.93	2904.3	2918.06	2916.4
2836.45	2835.62	2832.67	2840.14	2838.26
1459.65	1459.53	1460.73	1461.49	1426.85
1369.25	1368.65	1368.5	1369.69	1346.36
1348.31	1350.52	1361.22	1345.24	1340.13
1186.32	1185.43	1186.17	1187.52	1150.87
1111.13	1110.28	1111.03	1113.08	1086.39
1039.96	1040.71	1040.64	1041.09	974.5
687.26	686.95	687.09	687.61	769.1
471.52	470.97	469.82	472.78	441.22
333.46	335.37	333.1	334.23	355.95
263.19	259.98	257.79	266.83	288.13
252.25	255.73	253.03	265.5	277.46
251.6	252.05	252.6	251.95	208.64
161.34	160.37	188.69	158.95	180.73
145.03	156.7	160.32	138.55	143.37
139.05	140.98	140.09	126.8	139.86
79.57	79.2	89.44	86.02	66.22
18.58	20.65	53.19	18.17	23.66
10.7	13.92i	26.7	2.66i	20.49i
32.22i	23.59i	19.51i	21.53i	31.25i
87.04i	50.89i	36.33i	83.28i	57.98i

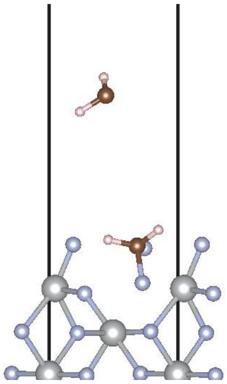

(1) pv 1

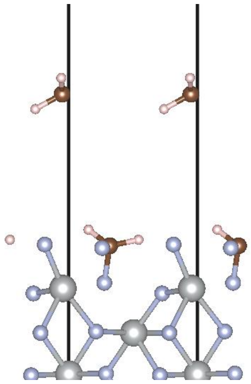

(2) pv 3

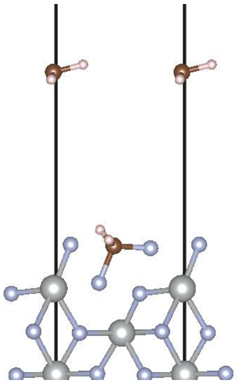

(3) pv 0

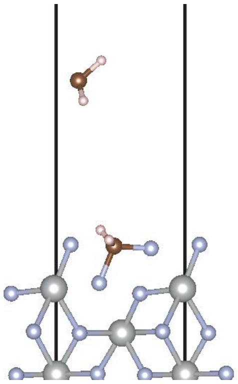

(4) pv 2

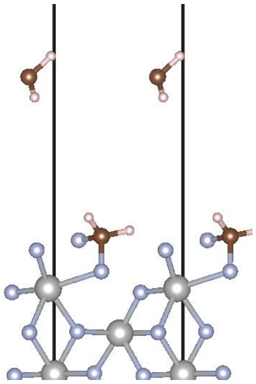

(5) pv 4

Figure S6: Structures after relaxation in group V.

7 Group VI

Table S21: Adsorption energies, and some selected structural parameters for the structures in group VI.

#	Orientation	Position	E_{ads}	d(CC)	d(CH)	d(CH)	d(CH)	d(CH)	d(NiF)	d(NiF)
1	p_2	4	-1.0811	1.367	1.087	1.094	1.087	1.094	1.862	1.862
2	p_1	0	-1.0609	1.357	1.090	1.092	1.090	1.092	1.823	1.898
3	p_2	3	-1.0200	1.358	1.092	1.094	1.092	1.094	1.809	1.809
4	p_1	4	-0.8176	1.355	1.089	1.092	1.089	1.092	1.747	1.747
5	f_2	3	-0.1788	1.354	1.086	1.088	1.086	1.088	1.987	1.987

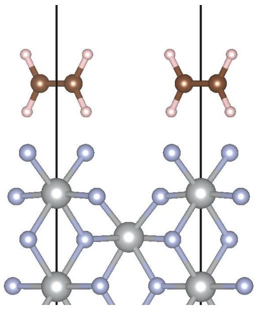

(1) p_2 4

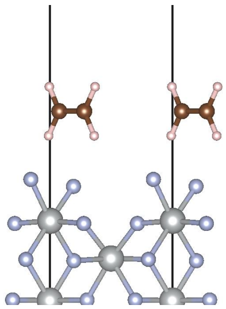

(2) p_1 0

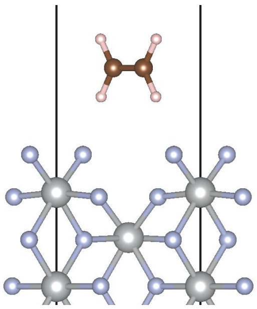

(3) p_2 3

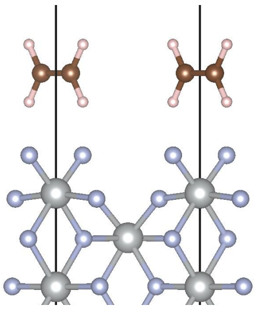

(4) p_1 4

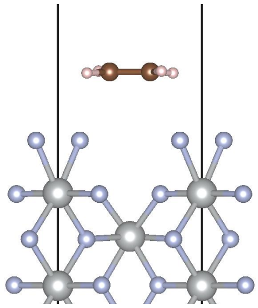

(5) f_2 3

Figure S7: Structures after relaxation in group VI.

Table S22: Magnetic moments on surface Ni, F and all atoms of ethene for group VI.

Ion/Position	Ion	Ion	Ni	F	F	C	C	H	H	H	H
1	p_2	4	-2.229	-0.375	-0.375	-0.069	-0.069	0.000	0.000	0.000	0.000
2	p_1	0	-2.235	0.046	0.210	0.047	0.047	-0.001	-0.002	-0.001	-0.002
3	p_2	3	-2.237	-0.356	-0.356	0.047	0.047	-0.002	-0.003	-0.002	-0.003
4	p_1	4	-2.161	-0.119	-0.119	0.052	0.052	-0.002	-0.002	-0.002	-0.002
5	f_2	3	-2.090	0.350	0.350	-0.040	-0.040	0.000	0.001	0.001	0.000

Table S23: Charge transfer on surface Ni, F and all atoms of ethene for group VI.

Ion/Position	Orientation	Position	Ni	F	F	C	C	H	H	H	H
1	p_2	4	0.006	0.205	0.224	-0.066	-0.032	0.012	-0.159	0.012	-0.159
2	p_1	0	-0.015	0.166	0.173	-0.047	0.003	-0.006	-0.109	-0.008	-0.118
3	p_2	3	-0.004	0.202	0.221	-0.043	0.035	-0.001	-0.143	-0.001	-0.143
4	p_1	4	-0.006	0.224	0.243	-0.064	0.047	-0.025	-0.123	-0.025	-0.123
5	f_2	3	0.094	0.093	0.101	-0.080	-0.019	-0.047	-0.049	-0.049	-0.047

Table S24: Vibrational frequencies for the structures in group VI.

4	0	3	4	3
p_2	p_1	p_2	p_1	f_2
3199.33	3186.61	3157.33	-	-
3183.06	3162.49	3128.04	-	-
3066.84	3078.0	3039.3	-	-
3052.77	3066.81	3038.41	-	-

Continued on next page

1521.14	1553.45	1546.08	-	-
1414.67	1412.78	1422.27	-	-
1309.83	1320.2	1326.92	-	-
1192.0	1203.73	1210.28	-	-
985.95	992.48	1002.98	-	-
937.49	952.43	961.9	-	-
821.51	810.3	825.35	-	-
719.29	737.23	632.01	-	-
390.84	423.68	439.94	-	-
331.54	257.16	381.24	-	-
269.64	187.94	201.37	-	-
240.73	168.38	172.59	-	-
229.25	148.89	151.94	-	-
162.06	129.51	145.07	-	-
155.78	102.21	132.16	-	-
108.76	91.34	104.18	-	-
89.01	81.02	85.21	-	-
39.81	55.75	79.0	-	-
47.94i	28.53i	21.59i	-	-
200.25i	38.0i	34.43i	-	-
